# Metagenomic analysis of fecal and environmental microbiota in rural mixed livestock farming systems in South Africa

**DOI:** 10.3389/fcimb.2026.1828785

**Published:** 2026-07-09

**Authors:** Thobeka Promise Mthembu, Nompilo Lucia Hlongwane, Adeola Salawu-Rotimi, Khanyisile Hadebe, Rian Pierneef

**Affiliations:** 1 Agricultural Research Council, Biotechnology Platform, Onderstepoort, Pretoria, South Africa; 2 Department of Biochemistry, Genetics and Microbiology, University of Pretoria, Pretoria, South Africa; 3 Department of Agriculture and Animal Health, College of Agriculture and Environmental Sciences, University of South Africa, Roodepoort, South Africa; 4 Inqaba Biotec, Pretoria, Gauteng, South Africa

**Keywords:** abundance, diversity, small-scale production, soil, water

## Abstract

In South African rural areas, farmers often practice mixed extensive livestock farming, facilitating microbial exchange among and between animal species and their environment. The composition and transmission potential of microbiomes between animals and their environments in these smallholder livestock systems remain largely unexplored, creating a gap in understanding how mixed-livestock farming affects gut and environmental microbiomes. Shotgun metagenomics was used to uncover the fecal and environmental microbiota in smallholder mixed livestock systems, aiming to understand microbiome transfer within these systems. A total of 111 samples were collected in KwaZulu-Natal and Eastern Cape provinces of South Africa, including 76 fecal samples from cattle, goats, sheep, pigs, and chickens; 18 soil samples; and 17 water samples. Taxonomic analysis of the sequencing data identified Proteobacteria as the dominant phylum across most hosts, except that pigs were dominated by Firmicutes. *Moraxellaceae* and *Pseudomonadaceae* were the differentiating families between monogastrics and ruminants. Although microbial diversity differences were significantly attributed to the host, genera such as *Acinetobacter*, *Chryseobacterium*, *Flavobacterium*, *Pedobacter*, and *Pseudomonas* were consistently found across all animal and environmental hosts. Cattle shared more genera with the environment than other animal species. Opportunistic pathogens, including *Enterococcus* spp., *Escherichia coli*, and *Clostridium* spp., were found across all the livestock species, and were highest in chickens. Additionally, some pathogens were detected in water but none in soil, suggesting water as a potential medium for pathogen transmission. The microbial exchange between livestock and their surroundings highlights the permeability of host-environment boundaries in smallholder systems.

## 1 Introduction

The United Nations’ Sustainable Development Goals (SDGs) aim to improve human livelihoods by ending poverty and reducing inequality among people. Agriculture is the foundation for achieving some SDGs, including SDGs 2 and 3, which target hunger, food insecurity, and malnutrition. Livestock farming is central to the response for enhancing food and nutrition security in marginalised households, serving as a mechanism to advance progress towards these SDGs (Danso-Abbeam et al., 2024; Mdiya et al., 2025; Oluwatayo, 2019; Xaba et al., 2025). In rural areas, smallholder farmers typically raise various types of livestock for multiple reasons, including financial investment, income generation, and consumption (Steinfeld and Mack, 1995). Successfully raising livestock for market purposes necessitates a significant investment of capital, as well as the availability of essential resources such as feed, water, and veterinary care. Additionally, sufficient space is crucial for the animals to thrive, along with a sufficient labor force to manage their daily needs (Giller et al., 2021; Oluwatayo, 2019). The smallholder farming sector is characterized by free-grazing systems with low production input and is practiced in ~ 1.4 million households in South Africa, with significant herds in the rural Eastern Cape and KwaZulu-Natal (Statistics South Africa, 2024). Many smallholder farmers face significant challenges in accessing critical resources, limiting their ability to profitably engage in livestock production. Livestock in smallholder farming systems are often prone to challenges such as insufficient nutritious feed, limited access to clean drinking water, extreme temperatures, and exposure to pathogens, all of which are related to unfavorable climatic changes (Eeswaran et al., 2022; Myeki and Bahta, 2021). Socio-economic conditions influence husbandry practices and have an impact on livestock welfare in smallholder farming systems (Enahoro et al., 2021; Hopker et al., 2021). Due to the nature of the smallholder livestock farming systems in rural areas, the animals are adapted to survive with minimal input from the farmer.

Animal health is important for performance and productivity. The interaction between genetic and environmental factors determines the health of an organism (Hu et al., 2020). The symbiotic microbial communities that are harbored by the guts of animals, namely the gut microbiota, also play a role in maintaining the health status of their hosts (El-Sayed et al., 2021; Pickard et al., 2017). The roles of the gut microbiota include the production of vitamins, metabolism, digestion of resistant starch, and pathogen exclusion (Jandhyala et al., 2015). The gut microbiota is shaped by the adaptability of an organism to different environmental factors (Gharechahi et al., 2021; Peixoto et al., 2021). Several studies have been carried out through “omics” technologies to explore animal gut microbiota and investigate the impact of host genetics, diet, and the environment in shaping the gut microbiota (Abbas et al., 2020; Dill-McFarland et al., 2019; Fan et al., 2020; Gharechahi et al., 2021; Li et al., 2019). The relationship between gut microbiome composition and traits such as feed efficiency, milk production, and egg production has been documented in animals (Elokil et al., 2020; Paz et al., 2018; Sofyan et al., 2019). Promising findings for improving animal health through modulation of the gut microbiota have been discovered in chickens, pigs, and steers (Chen et al., 2021b; Hu et al., 2018; Siegerstetter et al., 2018; Zhou et al., 2018). However, most of these studies have been conducted on experimental and commercial farms in developed countries, leaving a significant knowledge gap regarding the gut microbiomes of rural animals in developing regions (Forcina et al., 2022). Pylro et al. (2015) recommended integration of local microbiome research to create a robust International Microbiome Initiative. Furthermore, a global bias in ruminant microbiome datasets, with underrepresentation of regions such as Africa, was highlighted by Ortiz-Chura et al. (2024). Therefore, investigating microbiomes within specific environmental and production settings is essential for generating relevant and translatable insights. Furthermore, the implication of sharing the environment in gut microbiome transmission in mixed livestock systems is still underexplored.

Sequencing of marker genes such as 16S rRNA and internal transcribed spacer (ITS) region has been used to profile microbial communities due to their cost-effectiveness and well-established analytical pipelines (Caporaso et al., 2011; Knight et al., 2018; Schoch et al., 2012). The 16S rRNA gene contains the conserved and variable regions, which can be used to classify bacteria and archaea, while ITS is used for fungal classification due to its higher interspecific variability (Knight et al., 2018; Schoch et al., 2012). Although these approaches are cost-effective, they cannot capture full diversity and have limited taxonomic resolution and functional profiling (Hillmann et al., 2018; Quince et al., 2017). Shotgun metagenomics overcomes the limitations of amplicon sequencing, but the sequencing approach used depends on the study objectives (Quince et al., 2017). The application of shotgun metagenomics in livestock has provided high taxonomic resolution, enabling detailed characterization of the microbial diversity in the rumen contents of the South African mutton Merino sheep and the fecal samples of dairy cows from various regions (Akanmu et al., 2023; Khan et al., 2023). Additionally, a reference database was created to identify predicted genes and shared bacterial species between goats and sheep in a study utilizing shotgun metagenomics (Zhang et al., 2024). This research revealed significant differences in microbial composition based on rearing systems, highlighting the potential of high-throughput sequencing to elucidate real-world dynamics (Zhang et al., 2024). Furthermore, Kim et al. (2023) employed shotgun metagenomics sequencing to uncover taxonomic profiles and pathogenic risk markers in the feces of chickens and pigs. This method allows for the detection of microorganisms within a sample without the need for culturing, thereby identifying even those microorganisms that cannot be cultured (Durazzi et al., 2021).

The gut and environment host complex microbial communities that are often not accessible through traditional culturing techniques and can only be partially analyzed using amplicon sequencing. Therefore, shotgun metagenomic sequencing proves to be a valuable tool for studying these intricate microbial ecosystems. Shotgun metagenomics sequencing has been employed to explore the gut microbiomes of livestock such as cattle, chickens, goats, pigs, and sheep (Durazzi et al., 2021; Forcina et al., 2022; Kim et al., 2023; Tanca et al., 2017). Furthermore, microbial communities in soil, water, and air have been characterized using shotgun metagenomics (Maciel-Guerra et al., 2023; Mhuireach et al., 2022). Advances in interdisciplinary research have seen metagenomics, metabolomics, and metatranscriptomics employed to understand the interaction between gut microbiomes and their hosts (Li et al., 2019; Omondi et al., 2024). Adoption of new technologies such as microbiome profiling in smallholder farms is encouraged due to their potential to strengthen smallholder resilience and sustainability (Aina and Donaldson, 2025; Fontaine and Callens, 2025). Furthermore, the utilization of new technologies in small farms and developing countries promotes equity and inclusion of developing countries in research (Fontaine and Callens, 2025). This study utilizes shotgun metagenomics to investigate the gut microbiomes of livestock raised in rural mixed-livestock systems under extensive management. Specifically, it aims to (i) characterize and compare the taxonomic composition and diversity of cohabiting livestock species, (ii) profile microbial communities in associated environmental niches such as soil and water, and (iii) evaluate the impact of rearing mixed livestock species on gut microbial diversity and the potential overlap between host and environmental microbiota.

## 2 Materials and methods

### 2.1 Study design

**Figure 1:**
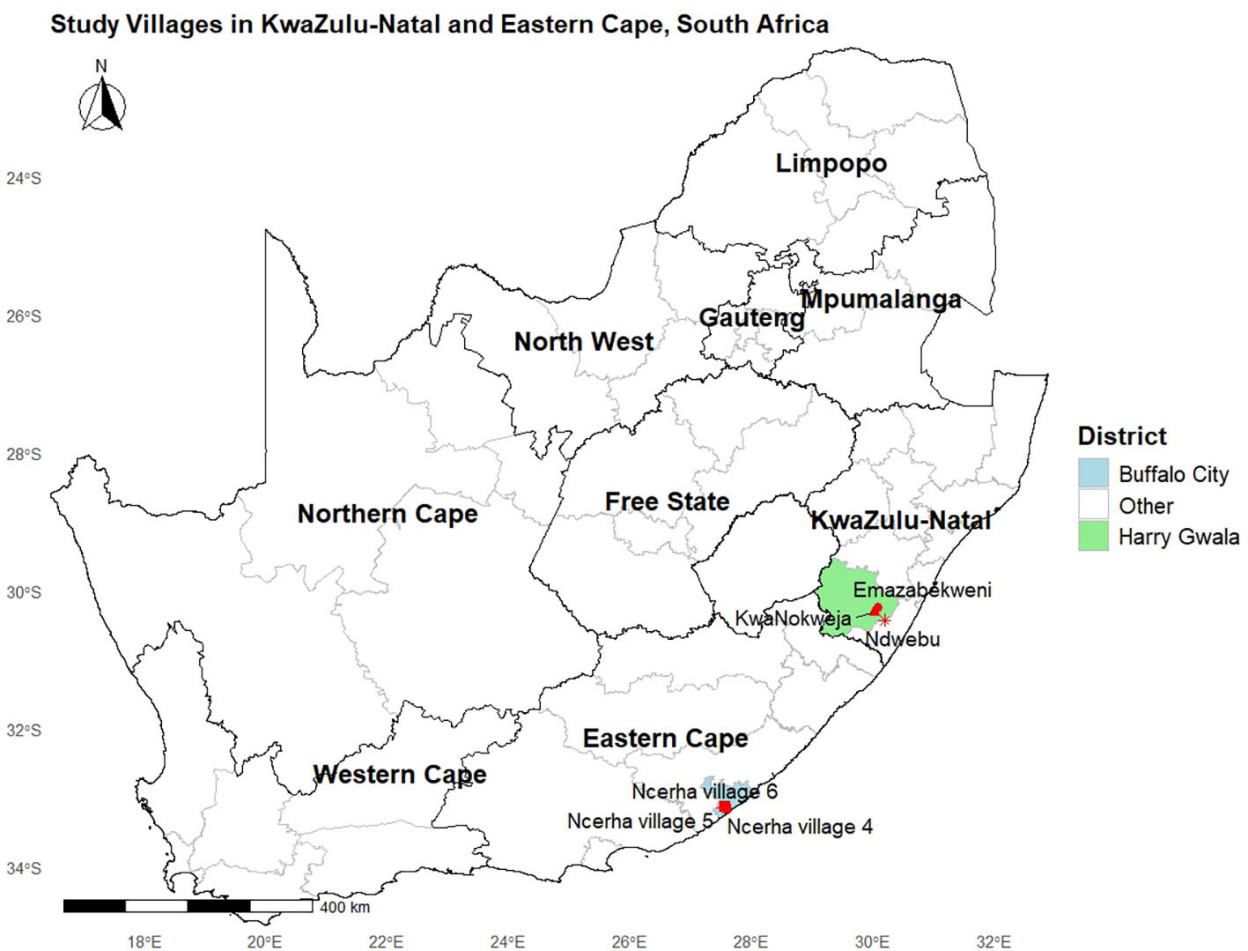
Map of South Africa showing study villages' locations in KwaZulu-Natal and the Eastern Cape.

A comparative cross-sectional metagenomics study was conducted in three villages in the Eastern Cape (Buffalo City Metropolitan Municipality: Ncerha villages 4, 5, and 6) and three villages in KwaZulu-Natal (Harry Gwala District: Emazabekweni, KwaNokweja, Ndwebu) (**Figure 1**). The villages in the Eastern Cape are ~3.72 kilometers apart, whereas the KwaZulu-Natal villages are ~18.07 kilometers apart. The Eastern Cape has the largest smallholder farming sector in South Africa, followed by KwaZulu-Natal. The University of Pretoria Animal Ethics Committee (NAS162/2023) and the Agricultural Research Council Onderstepoort Veterinary Institute Animal Ethics Committee (23.23) granted ethical approval for this study.

Households that kept multiple types of livestock species, including cattle, goats, chickens, sheep, and pigs, were visited to collect fresh fecal droppings from livestock, soil, and water samples. Consent was obtained from the farmers to gather baseline household data through a structured questionnaire survey and biological samples from their households. The number of samples collected per household was dependent on the types of livestock species available in a household, and the minimum criteria was three animal types per household. From each household, 1 fecal sample was collected per animal species, making 76 fecal samples (15 cattle, 19 chickens, 18 goats, 16 pigs, and 8 sheep) from all the animals in all households. Also, 1 soil and 1 water sample were collected from each household, totaling 35 (18 soil and 17 water samples). A metadata table showing the list of samples is attached (**Supplementary Table 1**).

Fresh, moist fecal droppings from each animal species were collected with gloves and placed into 50 ml centrifuge tubes. A new pair of gloves was used during each sample collection to avoid cross-contamination. Soil samples were collected from areas frequented by animals in the yards, such as near the troughs and walkways for animals. Surface soil was slightly scrapped and samples were collected at a depth of 0–10 cm below the surface using 50 ml centrifuge tubes. For water samples, 1L plastic bottles were submerged below the water surface in the animal drinking troughs to collect resident microbial communities. The samples were transported on ice to the Agricultural Research Council-Biotechnology Platform Core laboratory (Pretoria, South Africa) for processing.

### 2.2 DNA extraction and sequencing

Microbial DNA was extracted from 200 mg of fecal samples and 250 mg of soil samples using the ZymoBIOMICS DNA Miniprep kit (Zymo Research International). Two hundred and fifty ml of water samples were filtered through a 0.45 µm filter paper, and the filter paper was incubated in 5 ml PBS buffer. After incubation, 250 µl was used for cell lysis and DNA extraction. DNA concentration was assessed by Qubit fluorometer (Thermo Fisher Scientific), and the DNA was stored at -20 ^0^C. Libraries were prepared following the Universal DNA mini-prep library kit (MGI Tech Co., Shenzhen, China). Briefly, DNA samples were digested mechanically using the Covaris ultrasonicator to get 150 bp fragments, adaptors were ligated to the fragments, and paired-end sequencing was performed on the MGI DNBSEQ G400 platform (MGI Tech Co., Shenzhen, China).

### 2.3 Bioinformatics and statistical analysis

Analysis of the sequencing reads was conducted on KBase (Arkin et al., 2018). Data quality was assessed with FASTQC v0.12.0 (Andrews et al., 2010). High-quality data with phred scores above 30 were obtained; therefore, the trimming step was skipped (**Supplementary Figure 1**). Taxonomic classification was carried out using Kaiju v1.9.0 to identify the abundance of bacteria at various taxonomic levels with the RefSeq (no Euks) database specified as reference (Menzel et al., 2016). Tables of read counts for taxa per sample were generated on Excel and uploaded to R version 4.4.1 (R Core Team, 2024) (**Supplementary Tables 2**-**4**). For downstream analysis on R, a phyloseq (McMurdie and Holmes, 2013) object was created to merge the metadata with taxa for each sample. To generate the relative abundance table, the counts of reads were aggregated at the phylum level, and relative abundance was calculated by dividing each taxon count by the total reads in that sample. The table was converted into long format for the generation of plots. The outputs were analyzed using the R packages such as phyloseq (McMurdie and Holmes, 2013) and tidyverse (Wickham et al., 2019), implemented in R. Plots were generated and visualized using the ggplot2 package (Wickham, 2016).

Alpha diversity was assessed with Chao1, Shannon, Observed, and Simpson indices, while beta diversity was explored through Principal Coordinate Analysis (PCoA) using the Bray-Curtis dissimilarity matrix. Removal of low-abundant genera and normalization to account for differences in sequencing depths were done by DESeq2 (Love et al., 2014). The Kruskal-Wallis test was used to identify significant differences in alpha diversity between hosts, and *post-hoc* pairwise comparisons were done with Dunn’s test with P-values adjusted by the Benjamin-Hochberg method. PERMANOVA’s vegan package adonis2 function was used to assess the beta diversity differences, with a P-value threshold set at 0.05 (Oksanen et al., 2007). Differentially abundant microbial genera were identified by LEfSe (linear discriminant analysis Effect Size) in the microbiomeMarker package (Segata et al., 2011). For pathogen identification, the list of identified species was cross-referenced with the virulence factor database and the NCBI pathogen detection database to identify potential pathogens.

## 3 Results

The distribution of the samples across the sampled villages and livestock is shown in **Table 1**. The farmers in this study were mostly males (77%) and pensioners (36.4%) with various reasons for keeping livestock, including generating income (50%), financial investments (22.7%), and consumption (13.6%). In all households, animals shared water sources, with tap water stored in containers or troughs specifically for drinking. Additionally, 50% of the households reported that their animals also drank water from nearby rivers. Livestock in 54.5% of the households relied solely on natural grazing, while 41% also supplemented this with commercial feed. Furthermore, chickens and pigs were fed kitchen waste. The ruminants were herded to the fields for grazing, while chickens and pigs roamed around the yards, foraging for edible materials. All households had animal housing with natural soil floors and dry fecal matter, which was not cleaned regularly. Nearly all farmers used tetracycline antibiotics and antiparasitic medications, with a higher usage of medication in the Eastern Cape compared to KwaZulu-Natal. During the time of sample collection, none of the farmers reported having sick animals.

**Table 1:** The locations and numbers of samples collected from each animal and environmental host.

Province	Eastern cape			KwaZulu-Natal			Total per host
Village	Ncerha village 4	Ncerha village 5	Ncerha village 6	KwaNokweja	Ndwebu	Emazabekweni	
cattle	3	3	3	1	2	3	15
chicken	3	3	3	2	4	4	19
goat	3	3	3	2	4	3	18
pig	4	3	3	3	2	1	16
sheep	3	0	0	2	0	3	8
soil	3	3	3	3	4	2	18
water	3	3	3	3	2	3	17
Total per location	**22**	**18**	**18**	**16**	**18**	**19**	**111**

The bold values represent the number of samples collected from each village.

### 3.1 Microbial composition of fecal and environmental samples

**Figure 2:**
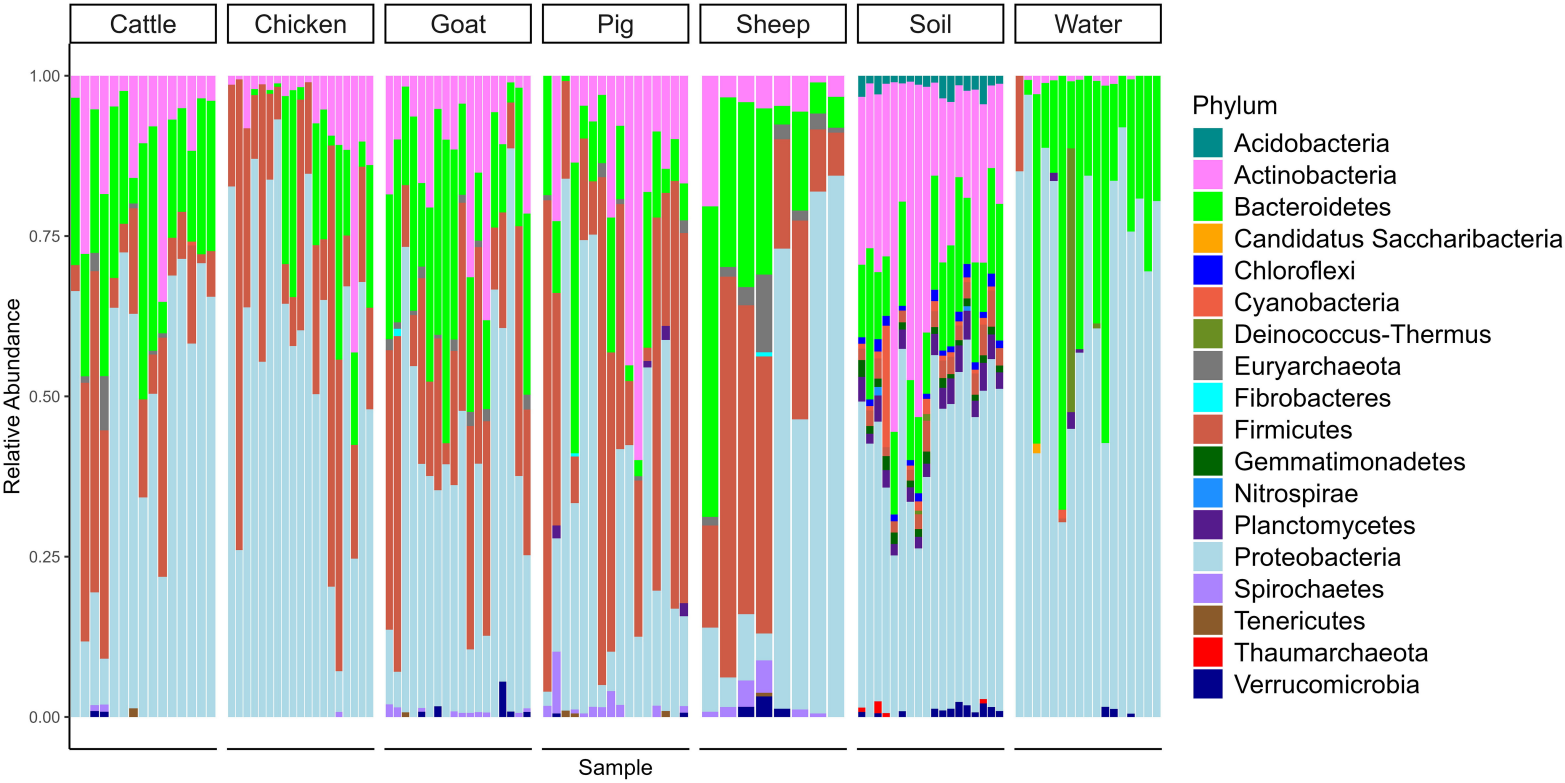
Relative abundance plot showing the microbial phyla in cattle, chickens, goats, pigs, soil, and water.

Taxonomic analyses identified 18 distinct phyla across all animal and environmental hosts (**Figure 2**). Among these, the most abundant were Proteobacteria, Firmicutes, Bacteroidetes, and Actinobacteria. Proteobacteria were most abundant in water (70.2%), followed by chickens (58.4%), cattle (49.5%), soil (46.3%), goats (36.4%), and least abundant in pigs (32.7%). Pigs were predominated by Firmicutes and had a higher abundance of Actinobacteria, while the other animal hosts were dominated by Proteobacteria and had a higher abundance of Bacteroidetes. In cattle, Bacteroidetes were the second most abundant, while in goats, chickens, and sheep, Firmicutes were in higher abundance than Bacteroidetes. Firmicutes were in low abundance in the soil and water compared to the animal hosts. Among the 18 phyla, 5 (26.3%) comprising Acidobacteria, Chloroflexi, Gemmatimonadetes, Thaumarchaeota, and Nitrospirae were exclusive to soil, indicating a high microbial diversity in soil compared to other hosts. Deinococcus-Thermus and Candidatus Saccharibacteria phyla were only present in water.

**Figure 3:**
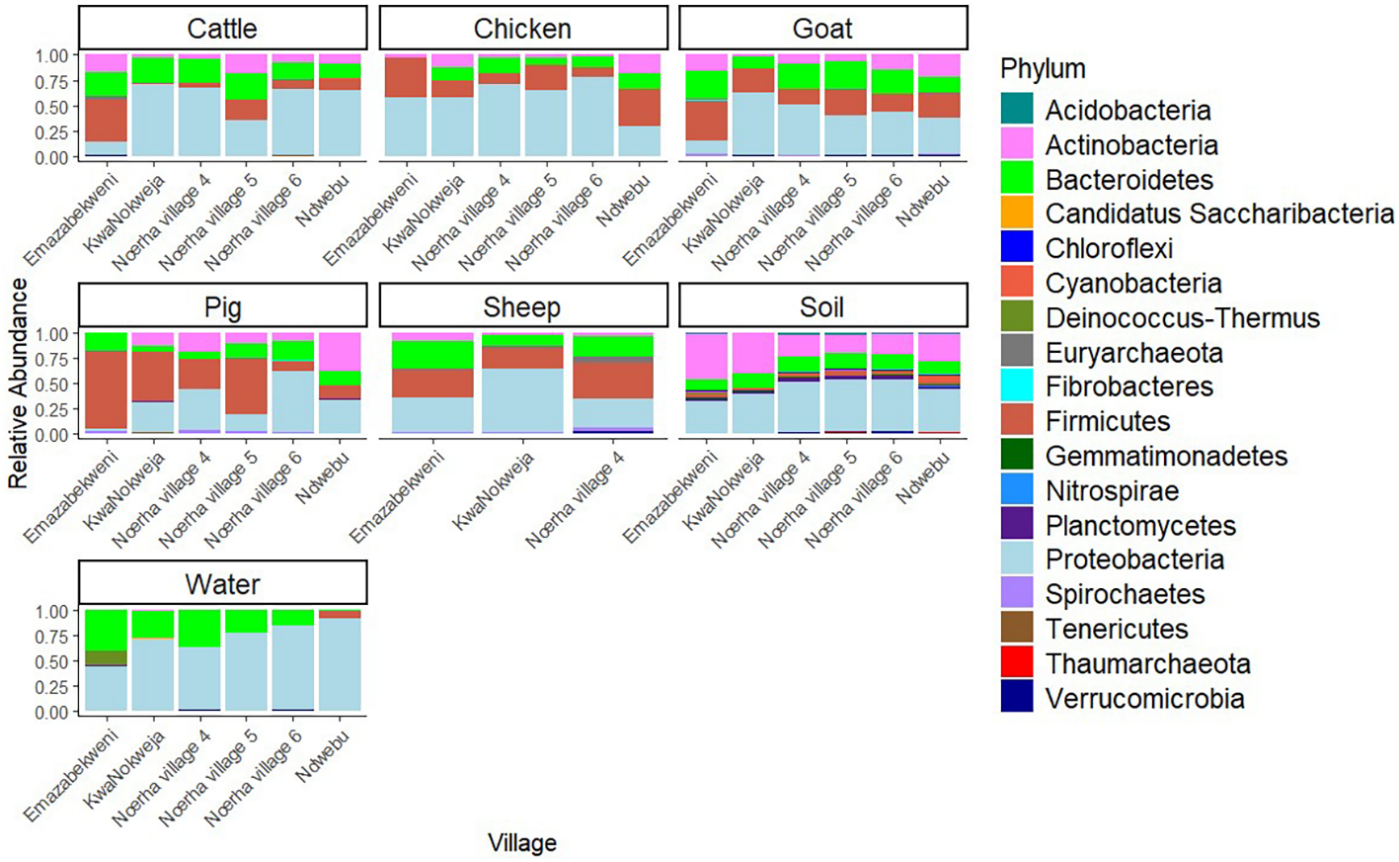
Phylum-level microbial composition for each host per village.

Village-specific variations were observed; cattle and goats from Emazabekweni were dominated by Firmicutes and had the lowest abundance of Proteobacteria compared to the other villages (**Figure 3**). Sheep from Ncerha village 4 had a higher abundance of Firmicutes than Proteobacteria. Firmicutes were in the highest abundance in chickens from Ndwebu, while Ncerha villages 4 and 6 presented a high enrichment of Proteobacteria and the lowest abundance of Firmicutes. Although Firmicutes primarily dominated pigs, Proteobacteria were most prevalent in Ncerha villages 4 and 6. Additionally, pigs from Ndwebu exhibited a distinct microbial community structure dominated by Actinobacteria. The soil samples from all villages had high proportions of Proteobacteria, followed by Actinobacteria and Bacteroidetes, except in KwaNokweja, where Actinobacteria were slightly more abundant than Proteobacteria. All the water samples were highly enriched with Proteobacteria; however, at a lower percentage in Emazabekweni (44.0%) compared to the other villages, which had higher than 50%. Firmicutes were only present in low abundance (0.7%) at Ndwebu.

**Figure 4:**
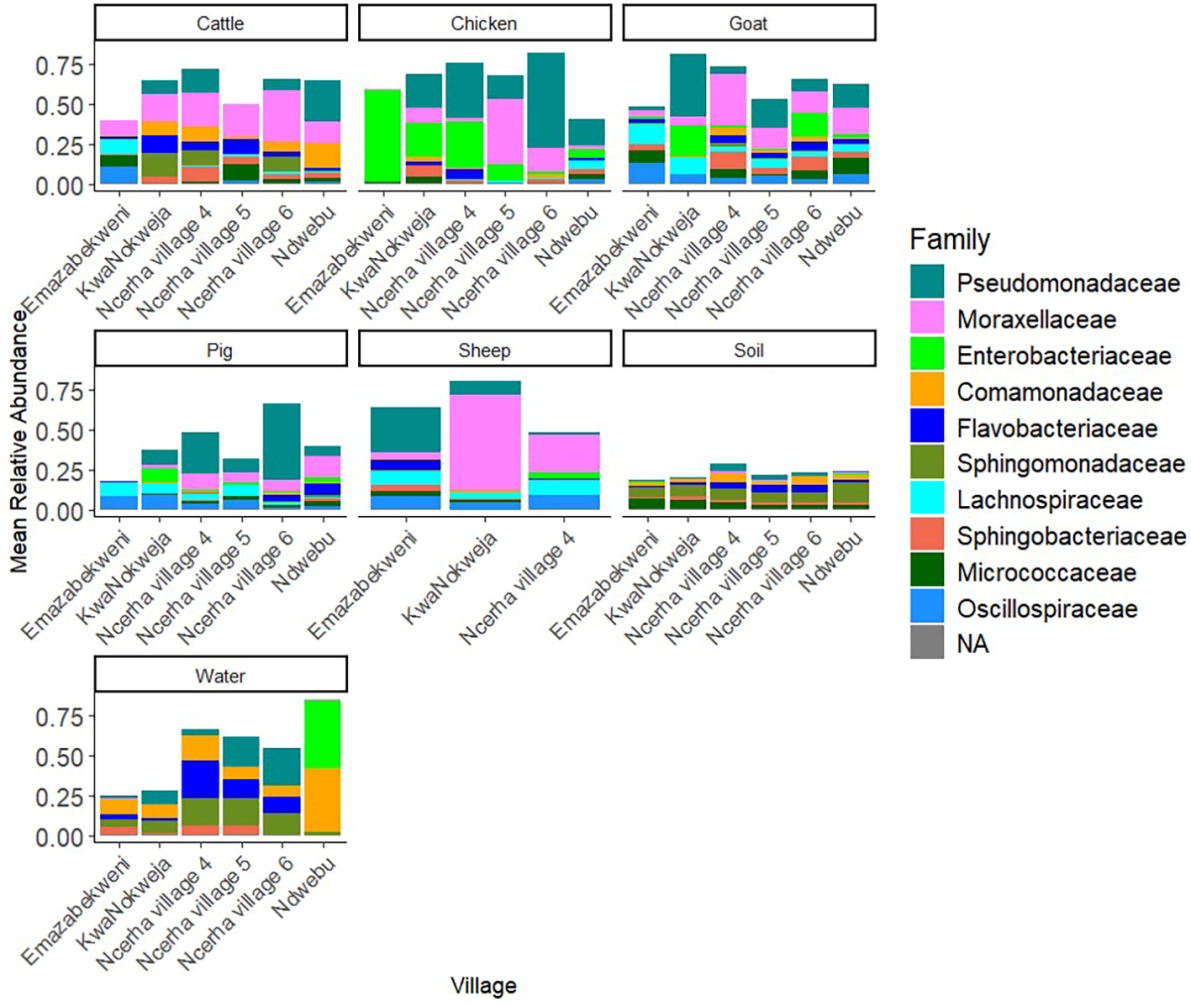
The distribution of the top 10 microbial families across hosts in each village.

The samples were further classified into 152 families, 305 genera, and 510 species. Overall, at the family level, *Moraxellaceae* were predominant in cattle, goats, and sheep, while chickens and pigs were primarily associated with *Pseudomonadaceae* (**Figure 4**). Although goats and sheep had a high prevalence of *Moraxellaceae*, they also exhibited high levels of *Pseudomonadaceae* compared to cattle. Village-specific variations were also observed, and significant differences were denoted by PERMANOVA (P < 0.05) in water and chicken samples (**Table 2**). Cattle from Emazabekweni village, KwaZulu-Natal, had microbial families enriched with *Oscillospiraceae*, *Lachnospiriaceae*, and *Micrococcaceae*, unlike the other villages, which were dominated by *Moraxellaceae* followed by *Pseudomonadaceae*. Similar to cattle, goats in Emazabekweni were enriched with *Oscillospiraceae*, *Lachnospiriaceae*, and *Micrococcaceae*, while the other villages had high levels of *Moraxellaceae*, *Pseudomonadaceae*, and *Enterobacteriaceae*. Differences were also observed in sheep from Emazabakweni, which were mainly composed of *Pseudomonadaceae*, with very low levels of *Moraxellaceae*, which largely characterized the microbial communities in sheep from other villages. The family *Enterobacteriaceae* was prevalent in chickens from Emazabekweni, Ncerha village 4, and KwaNokweja, while *Pseudomonadaceae* showed a strong presence in Ncerha villages 4 and 6. Ncerha village 5 had a high proportion of *Moraxellaceae*, which appeared in low percentages in the other villages. Interestingly, families such as *Sphingomonadaceae*, *Comamonadaceae*, and *Flavobacteriaceae* showed microbial similarities among cattle, water, and soil.

**Table 2:** PERMANOVA results showing significant differences in microbial community composition of chicken and water samples (family level) from different villages.

Host	R^2^	F-value	P-value
Cattle	0.39004	1.5986	0.066
Goats	0.35181	1.3026	0.167
Chicken	0.44983	2.1258	0.01*
Pigs	0.39581	1.3102	0.135
Sheep	0.36403	0.7632	0.707
Water	0.50836	2.2748	0.001*
Soil	0.37929	1.4665	0.089

*significant (p < 0.05).

*Pseudomonas* and *Acinetobacter* were the most abundant genera. *Pseudomonas* was particularly abundant in chickens (25.2%) and pigs (22.6%), while *Acinetobacter* was abundant in sheep (28.6%), cattle (23.2%), and goats (17.6%). While *Acinetobacter* primarily colonized cattle in all the villages, goats from KwaNokweja and Ncerha village 5 were mainly colonized by *Pseudomonas*, whereas *Bacteroidetes* and *Allistipes* were the most abundant in Emazabekweni. Furthermore, in sheep samples, Emazabekweni was dominated by *Pseudomonas*, while *Acinetobacter* was the highest in all the other villages. Within the pig samples, *Lactobacillus*, *Solibacillus*, and *Rhodococcus* were in the highest abundance in Emazabekweni, Ncerha village 5, and Ndwebu, respectively. Chickens from Ncerha village 5 were highly enriched with *Psychrobacter*, while *Escherichia* and *Lactobacillus* were high in Emazabekweni. *Sphingomonas* was prevalent in soil samples; however, samples from Ncerha village 5 and KwaNokweja were mainly enriched with *Bradyrhizobium* and *Norcadioides*. Water samples varied in their microbial composition between villages, with genera such as *Pseudomonas*, *Polynucleobacter*, *Deinococcus*, *Flavobacterium*, and *Limnohabitans* predominating in different villages. Water from Ndwebu showed higher levels of gut-associated bacteria like *Escherichia* and *Lactococcus*.

### 3.2 Diversity analysis

**Figure 5:**
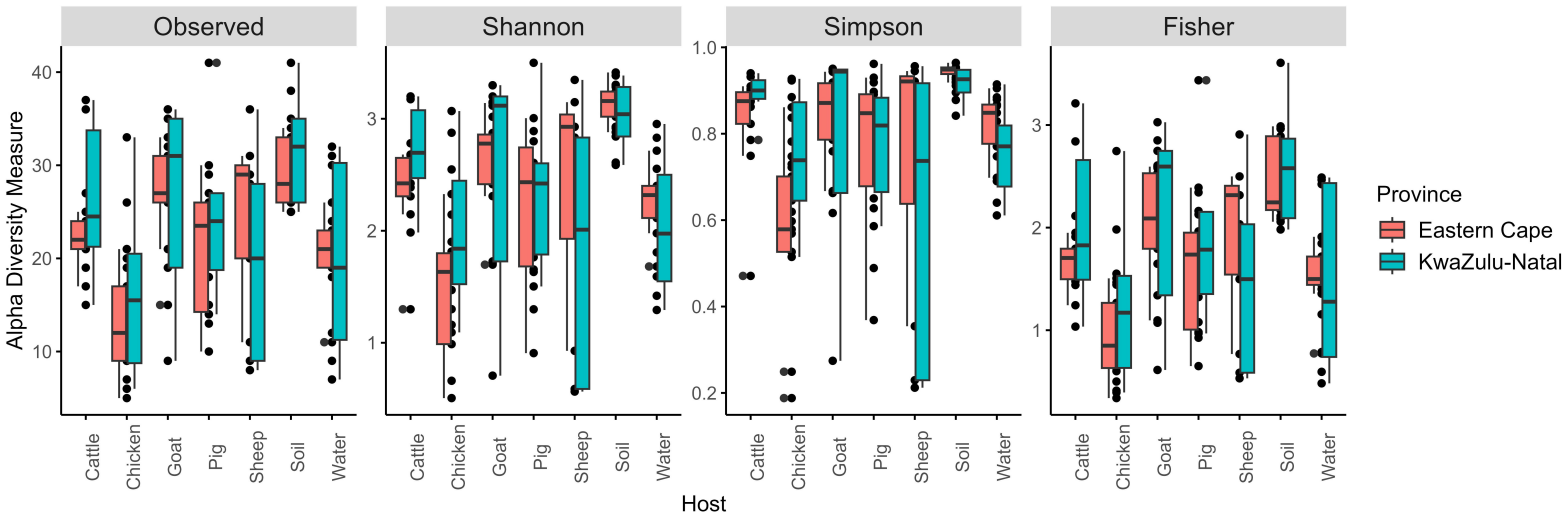
Boxplots illustrating alpha diversity in cattle, chicken, goat, pig, soil, and water microbiota using Observed, Shannon, Simpson, and Fisher indices in mixed livestock farming systems. The colors of the boxplots represent the provinces.

Alpha diversity was assessed based on Observed species richness, Shannon index, Simpson index, and Fisher’s alpha index to reveal microbial richness, evenness, and dominance at the genus level (**Figure 5**). Kruskal-Wallis rank sum test denoted significant differences in microbial diversity (P < 0.05) at all the diversity measures. Pairwise comparisons, assessed with Dunn’s test, showed significant differences, particularly between the soil and the other hosts (**Supplementary Table 5**). Soil consistently showed higher diversity than chickens, pigs, sheep, cattle, and water (P < 0.05). Among animal hosts, goats exhibited greater microbial community diversity, while the microbial diversity in chickens was significantly lower than that of cattle and goats. Although goats did not differ significantly from cattle, sheep, and pigs, the diversity of microbial communities among the latter displayed more similar trends. When comparing microbial diversity among hosts in each province, the Eastern Cape (P = 0.00004) showed more significant differences than KwaZulu-Natal (P = 0.04). Analysis of host types at the village level revealed that cattle from Emazabekweni exhibited greater diversity than cattle from Ncerha village 6 and Ndwebu, based on Observed and Fisher diversity indices.

**Figure 6:**
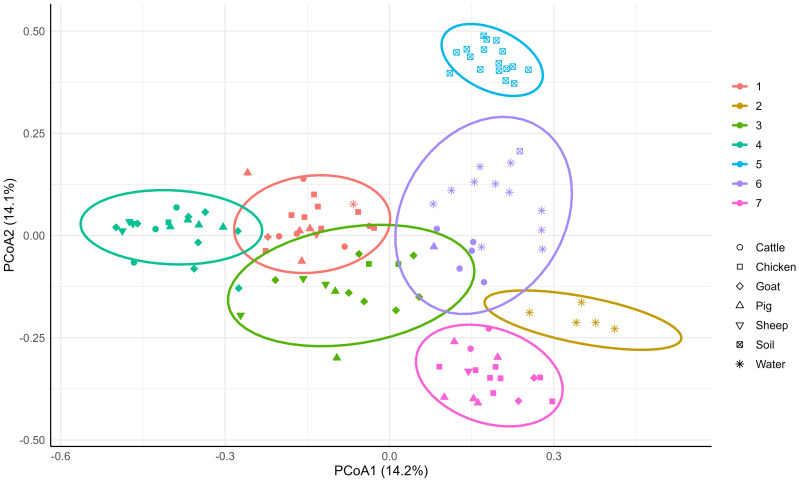
A principal coordinate analysis (PCoA) was performed to examine the microbial diversity among cattle, chickens, pigs, sheep, and goats that coexist in the same environment, as well as the microbiota present in the soil and water.

Beta diversity (**Figure 6**) showed a clear separation of soil samples in cluster 5 from the other hosts. Water samples were found in separate clusters 2 and 6, which were located close to each other, revealing some differences in microbial composition. Most of the water samples from KwaZulu-Natal were grouped closely together in cluster 6. In cluster 2, the water samples included those from Ncerha villages 5 and 6, along with one sample from KwaNokweja in KwaZulu-Natal. Cattle samples were also present in cluster 6, indicating a similar microbiota between the animal hosts and the water. Clusters 1, 3, 4, and 6 overlapped and consisted of animal hosts, which were distributed across clusters 1, 3, 4, 6, and 7. The chicken samples were distributed across clusters 1, 3, and 7, except for one sample from Ndwebu, which was located in cluster 4. All the chicken samples from Ncerha village 6 were in cluster 7, while chicken samples from Emazabekweni were in cluster 1. This significant difference was demonstrated by PERMANOVA (R^2^ = 0.48258, P-value = 0.027). None of the animal hosts formed a distinct cluster, which implies that there were commonalities in microbial composition among the livestock. Though the soil samples generally clustered together, those from Ncerha village 4 showed more similarity to samples from the KwaZulu-Natal villages. In contrast, the samples from Ncerha villages 5 and 6 were more closely related to each other. PERMANOVA analysis revealed that overall, 29.1% of the variation in microbial composition was significantly associated with host type (P < 0.05). The province and village showed statistically significant, but relatively weak differences in microbial composition with R² values of 0.02108 and 0.07143 and P-values of 0.006 and 0.003, respectively. However, these differences were not strong enough to result in distinct clustering by province or village. The microbial communities in water and chickens differed significantly between the provinces. Feed type, grazing versus grazing with supplementation, also had a modest impact on the composition of the gut microbiomes (PERMANOVA, R^2^ = 0.44, P = 0.02). There were no significant differences between animals that only drank water from the troughs and the animals that drank from the river (PERMANOVA, R² = 0.02, P = 0.1).

### 3.3 Shared and unique microbial genera among the animal hosts

**Figure 7:**
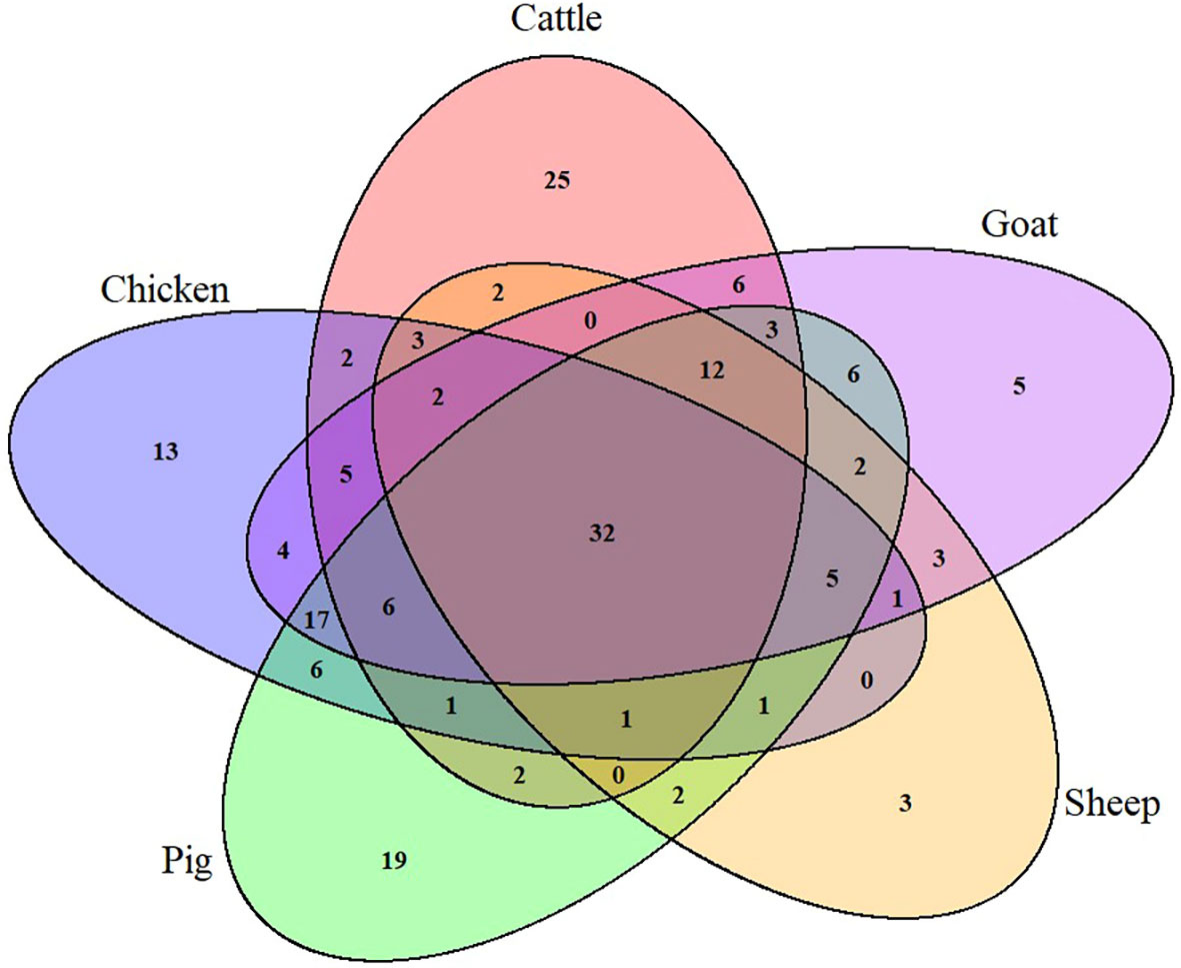
A Venn diagram of the shared and unique microbial genera present in animal hosts. Each circle represents the animal host. The numbers in overlapping circles indicate the shared microbial genera, and the numbers in the non-overlapping areas represent the unique microbiota.

Among the 305 microbial genera identified across all hosts, 189 (61.9%) were found in animals, with 32 genera shared by all the animal hosts (**Figure 7**). Cattle had 25 unique genera, followed by pigs and chickens with 19 and 13 genera, respectively. Chickens, goats, and pigs shared 17 genera, and followed by cattle, goats, pigs, and sheep, which shared 12 genera.

Analysis of the consistently present genera revealed *Pseudomonas* as the main genus across all hosts and *Clostridium* as one of the key genera, along with *Bacteroides*, *Acinetobacter*, and *Prevotella* in animal hosts. Goats and sheep showed consistent presence of *Alistipes*, *Lachnoclostridium*, *Methanobrevibacter*, and *Ruminococcus*, which were not found in soil and water. On the other hand, *Arthrobacter*, *Comamonas*, *Sphingobacterium*, *Flavobacterium*, and *Chryseobacterium* were persistent in goat and cattle samples and in the environmental samples.

### 3.4 Differentially abundant microbial genera in hosts

**Figure 8:**
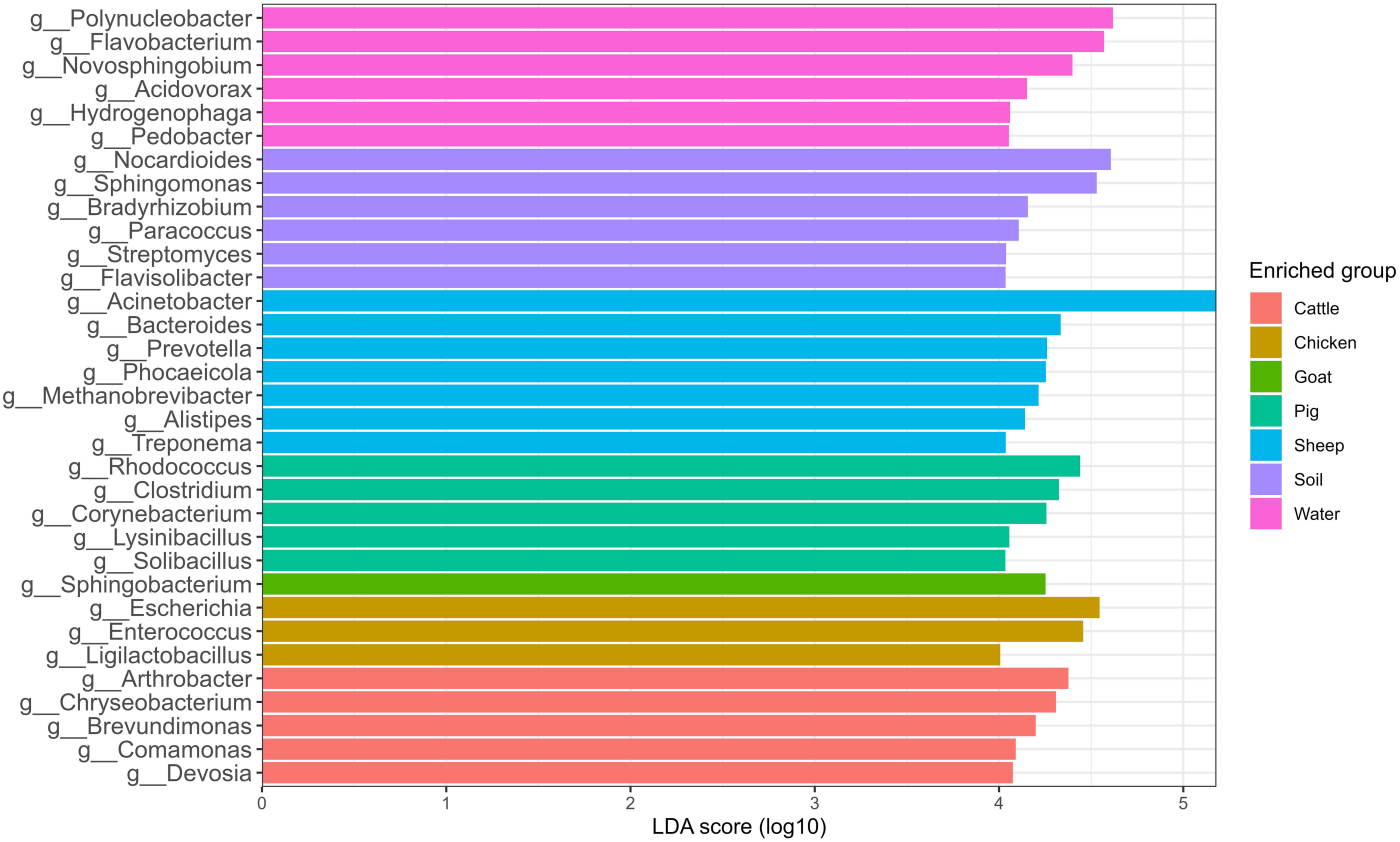
Linear discriminant analysis (LDA) performed by LEfSe (Linear Discriminant Analysis Effect Size) shows microbial genera with significantly different abundances in hosts.

The Linear discriminant analysis (LDA) with effect size (LefSe) identified the differentially abundant microbial genera across hosts (**Figure 8**). Members of Alphaproteobacteria (*Novosphingobium*), Betaproteobacteria (*Polynucleobacter*, *Acidovorax*, *Hydrogenophaga*), and Bacteroidetes (*Flavobacterium*, *Pedobacter*) were differentially abundant in water samples. The microbiota of soil was significantly enriched with genera of Alphaproteobacteria (*Bradyrhizobium*, *Sphingomonas*, *Paracoccus*), Actinobacteria (*Nocardioides*, *Streptomyces*), and Bacteroidetes (*Flavisolibacter*). Sheep showed variation in abundance of *Acinetobacter* (Gammaproteobacteria), *Methanobrevibacter* (Euryarchaeota), *Treponema* (Spirochaetes), and some members of the phylum Bacteroidetes. The marker genera in pigs were from the phyla Actinobacteria (*Rhodococcus*, *Corynebacterium*) and Firmicutes (*Lysinibacillus*, *Solibacillus*, *Clostridium*). Chickens also had a high enrichment of Firmicutes (*Enterococcus*, *Ligilactobacillus*) and Gammaproteobacteria (*Escherichia*). The microbial communities of cattle showed distinct profiles of Alphaproteobacteria (*Brevundimonas*, *Devosia*), Betaproteobacteria (*Comamonas*), Actinobacteria (*Arthrobacter*), and Bacteroidetes (*Chryseobacterium*).

### 3.5 Detection of potential pathogens

Low percentages of pathogenic species, as well as opportunistic pathogens of clinical importance, were detected in various livestock (**Table 3**). While the pathogens were distributed across almost all animal hosts, chickens had the highest enrichment, while in cattle, only *Paeniclostridium sordellii* was found in two samples from Emazabekweni. Moreover, pathogen detection in sheep, like cattle, was very low. *Enterococcus faecium*, *Escherichia coli*, and *Flavobacterium psychrophilum* were found in the water samples, indicating that water may serve as a medium for transmitting pathogens between animals. On the other hand, no pathogens were detected in the soil. Among the Eastern Cape villages, Ncerha village 4 had the highest pathogen detection rate, while village 6 had the lowest. In KwaZulu-Natal, pathogens were distributed across all villages.

**Table 3:** A list of human and livestock pathogens with their hosts and locations.

Species	Host	Province	Location
Clostridium botulinum	Goat	EC	Village 4
Clostridium perfringens	Chicken	EC, KZN	Village 4, village 5, Ndwebu, Emazabekweni
Paeniclostridium sordellii	Cattle	KZN	Emazabekweni
Enterococcus faecalis	Chicken Pig	EC, KZN KZN	Village 4, Ndwebu, Emazabekweni KwaNokweja
Enterococcus faecium	Chicken Pig Water	EC, KZN KZN KZN	Village 4, Ndwebu KwaNokweja Ndwebu
Escherichia coli	Goat Chicken Pig Sheep Water	EC, KZN EC, KZN KZN EC KZN	Village 4, village 6, KwaNokweja Village 4, village 5, Ndwebu, KwaNokweja, Emazabekweni KwaNokweja Village 4 Ndwebu
Shigella flexneri	Chicken	EC, KZN	Village 4, Emazabekweni
Enterococcus hirae	Chicken Goat Pig	EC, KZN EC EC	Village 4, Emazabekweni Village 4 Village 5
Aeromonas salmonicida	Goat	EC	Village 5
Flavobacterium psychrophilum	water	EC	Village 4
Stenotrophomonas maltophilia	Pig	KZN	Ndwebu
Enterococcus cecorum	Chicken	EC	Village 6
Ruminococcus gnavus	Chicken Goat Sheep Pig	KZN KZN KZN KZN	Ndwebu KwaNokweja KwaNokweja Emazabekweni

## 4 Discussion

Smallholder farmers in rural areas often rear animals under extensive production with minimal input, relying on their adaptability to thrive in prevailing environments. These farmers typically keep indigenous breeds or crossbreeds capable of enduring harsh conditions, including poor quality pasture, high temperatures, drought, and (Eeswaran et al., 2022; Forcina et al., 2022; Myeki and Bahta, 2021). This production system may influence the gut microbiome composition and transmission between the animals and the environment. Similar microbial communities and improved microbial diversity have been reported in mixed livestock that are reared in extensive systems (Qin et al., 2022; Song et al., 2020). Due to the increasing interest in studying the gut microbiome and the factors that influence its composition, researchers have begun to investigate how it is transmitted between organisms in social contact or sharing the same environment (Raulo et al., 2024; Sarkar et al., 2020). A study conducted by Zhang et al. (2022a) reported gut microbiome transmission in pigs and goats that shared the same pen. Van Gompel et al. (2020) found similarities between the gut microbiomes of farm workers and pig gut microbiomes compared to samples from humans who were not exposed to animals. The transmission of the gut microbiome is affected by microbial physiology, the ability to endure in the environment, and the capacity to survive and colonize a new host (Browne et al., 2017). One of the contributors to microbial transmission in farms is the environment through soil, water, and surrounding surfaces. A significant concern with sharing the environment is the ease with which pathogens can be transmitted between different hosts, presenting health risks. However, on a positive note, sharing the microbiome can have beneficial effects, as organisms may share advantageous microbiota (Zhu, 2023). This study investigated the fecal and environmental microbiomes of cattle, pigs, goats, sheep, and chickens coexisting within the mixed livestock systems in rural households.

The farms visited were operated mostly by males, pensioners, self-employed individuals, or those reliant on livestock sales, reflecting the demographics of rural smallholder livestock farmers. This demographic composition may influence management strategies for livestock, which in turn could impact the animals’ gut microbiomes. Raising livestock requires capital, and financial constraints in the smallholder sector often force animals to rely heavily on natural grazing and exposure to environmental microbiomes. Some farmers do provide supplementary feed, but financial limitations prevent them from supplying enough to meet the animals’ full nutritional requirements. The production systems in these households demonstrated a significant interaction between the animals and their environment, as the livestock housing structures were primarily made of natural soil, continually exposing the animals to environmental microbiota. Furthermore, different animal species shared the same water troughs in each household, which could facilitate microbiome transfer among the animals. Although the farmers indicated that they do use antibiotics, these are administered only for therapeutic purposes for a certain period.

The most dominant phyla observed were Proteobacteria, Bacteroidetes, Firmicutes, and Actinobacteria. These phyla have been reported to be the core gut microbial phyla in most studies; therefore, our results were in accordance with previous studies (de Carvalho et al., 2022; Domínguez et al., 2022; Elokil et al., 2020; Torres Manno et al., 2023). However, while the same phyla were present in this study, their relative abundances differed. Previous studies often reported higher abundances of Firmicutes or Bacteroidetes and lower levels of Proteobacteria (Elokil et al., 2020; Maake et al., 2021; Mootane et al., 2024).

The gut microbiomes of cattle from other countries in Africa were reported to be dominated by Firmicutes followed by Bacteroidetes (Aboshady et al., 2024; Mtshali et al., 2022; Omar et al., 2024; Wilkinson et al., 2020). Cattle from Tanzania, however, were predominated by Proteobacteria (Omar et al., 2024), which is in accordance with the results of the current study. Firmicutes and Bacteroidetes were also predominant in the gut microbiomes of chickens, as reported by other studies (Gilroy et al., 2021; Glendinning et al., 2024; Mootane et al., 2024). Furthermore, the predominance of Firmicutes and Bacteroidetes was observed in pigs and goats (Correa-Fiz et al., 2019; Maake et al., 2021; Rabapane and Matambo, 2024; Wang et al., 2021). In a study by Elokil et al. (2020), Firmicutes and Bacteroidetes were found to be in high abundance in high egg-producing chickens, while low egg-producing chickens were enriched with Proteobacteria. Research on the microbiomes of soil from a chicken farm, paddock soil, and faecal and dust samples from horses and cattle housed together revealed a predominance of Proteobacteria (Lim et al., 2020; Maciel-Guerra et al., 2023; Mhuireach et al., 2022; Park and Kim, 2019). This finding aligns with the results of the current study. Proteobacteria are responsible for preparing the gut for colonization by strict anaerobes, and their high abundance has been reported in the early stages of animal growth (Shin et al., 2015; Wilson, 2005). The high prevalence of Proteobacteria in mature animals has been associated with dysbiosis or gut inflammation, as many members of this phylum are opportunistic pathogens (Shin et al., 2015). This raises concerns in the current study as the fecal samples were collected from mature livestock, suggesting that the high abundance of Proteobacteria may indicate the presence of pathogens and an imbalance in the gut microbiome (Shin et al., 2015).

A healthy gut microbiome is characterized by a high abundance of Firmicutes and Bacteroidetes, which comprise approximately 90% of the gut microbiota, and a low abundance of Proteobacteria (Forcina et al., 2022). A high abundance of Proteobacteria has been linked to high grain diets, gut inflammation, less feed-efficient animals, and poor growth performance, which are expected outcomes for animals facing challenges in the smallholder farming sector (Myer et al., 2015; Shin et al., 2015; Zhang et al., 2022b). However, Proteobacteria are also reported as part of the baseline community composition and play roles in metabolic processes (Aruwa et al., 2021; Li et al., 2020). Several studies highlight the role of Firmicutes in fiber degradation, volatile and short-chain fatty acids production, aiding hosts in energy metabolism and nutrient absorption (Aruwa et al., 2021; Guerra et al., 2022; Henderson et al., 2015; Huang et al., 2023; Li et al., 2021a; Petri et al., 2013). Bacteroidetes are efficient at breaking down complex non-fibrous carbohydrates, starch, and protein, producing volatile fatty acids that provide energy to the host (Chang et al., 2020; Ge et al., 2023; Khan and Chousalkar, 2021; Li et al., 2021b). Actinobacteria are the least abundant in the gut, but they also play important roles in fiber degradation and gut homeostasis (Binda et al., 2018; Tan et al., 2014). Although Proteobacteria dominated most of the microbiomes, there were some village-driven differences, with Firmicutes dominating in some animal hosts from different villages. These differences can be partly attributed to the husbandry practices of the households in each village.

The observed differences in phylum composition, with pigs having a higher abundance of Firmicutes and Actinobacteria, while the other animal hosts showed higher abundances of Bacteroidetes, were due to the gut anatomy and feeding patterns of the animal hosts. Cattle, goats, sheep, and chickens feed on plant-based material, resulting in higher abundances of Bacteroidetes in their guts. This is supported by the results of a study by Schnorr et al. (2014), which found higher Bacteroidetes and lower Firmicutes and Actinobacteria in Hadza hunter-gatherer individuals who relied on plant-based foods. Pigs depend on starch-based feed, usually kitchen waste produced by households, although they also scavenge for food. The large ceca in chickens, which are absent in pigs, provide a niche for Bacteroidetes, explaining the higher abundance of Bacteroidetes in chickens compared to pigs, even though these animals are both monogastric (Nahm, 2006). The abundances of *Moraxellaceae* and *Pseudomonadaceae* differentiated between ruminants and monogastric animals. The mentioned bacterial families were found in very low abundances in the environment, indicating that they were specialized in the gut and their abundances were due to animal host physiology. A high abundance of *Moraxellaceae* was reported in warthogs compared to domestic and commercial pigs in a study conducted by Correa-Fiz et al. (2019). Another study reported a high abundance of *Moraxellaceae* in feces of cows from dairy farms (Du et al., 2020). *Moraxellaceae* and *Pseudomonadaceae* are regarded as environmental microbes; however, their dominance in animal samples in this study requires further investigation.

Alpha and beta diversity analyses revealed a distinct composition of the soil microbiome compared to animal microbiomes. Although the water samples clustered with some samples from animal hosts, they still showed some form of separation from the animal hosts. The differences in the microbiota of soil, water, and animal hosts can be explained by the ecological conditions of these niches, including temperature, oxygen, nutrient availability, and pH (Boyd, 2019; Pereira and Berry, 2017; Pietikäinen et al., 2005; Wei, 2020). Habitat preference among microorganisms plays a crucial role in determining which species dominate in various environments. For instance, habitat specialists found in soil prefer biogeochemical cycling, whereas gut specialists are strict anaerobes that can adapt to different pH levels, utilize mucin, and tolerate bile salts (Ma et al., 2025). In contrast, habitat generalists exhibit metabolic flexibility, stress tolerance, tolerance to bile salts, and genome plasticity, which enable them to survive in diverse environments (Ma et al., 2025). The gut is an anaerobic warm environment, supporting the growth of strict anaerobes and facultative anaerobes, while soil and water surfaces support the growth of aerobic microorganisms; however, stratified water bodies are anaerobic at higher depths, allowing for facultative anaerobes to thrive (Boyd, 2019; Karasova et al., 2022). Notable was the consistency of microbial composition in soil samples from different villages, while water samples had significant differences. The differences observed in the water samples were primarily due to the significant variation in water from Ncerha village 6 when compared to all the KwaZulu-Natal villages. This suggests that there are external influences on the microbial composition of water (Loucif and Chenchouni, 2024; Montiel-Molina et al., 2023). All water samples were collected from containers that are reserved for animal drinking in the yards. Hygiene practices towards water containers and the frequency of changing water could influence the microbiota of water from each household.

While beta diversity indicated significant differences in microbiota between animal and environmental samples, certain microbial genera were still shared between the two. Further analysis of the microbiota at the genus level revealed that the gut microbiota of ruminants shares a stronger resemblance with environmental microbiota compared to monogastric animals. Among the ruminants, cattle and goats have more environmental microbiota than sheep, possibly because cattle are large ruminants and goats are browsers compared to sheep, which are grazing small ruminants (Samuels et al., 2016). Browsers interact more with the environment by feeding on shrubs and climbing trees, and are exposed to more feeding sources, while grazers prefer grass (Mohammed et al., 2020). Although environmental microbiota can be found in the guts of animals, these are usually transient colonizers and not part of the core gut microbiota, therefore functional profiling could reveal the roles of these microbes in the guts (Lee et al., 2024). Gut retention time could also influence colonization by environmental microbiota. The gut retention time is longer in cattle, sheep, goats, pigs, and chickens, in that order (Schlecht et al., 2007; Svihus and Itani, 2019; Tsiplakou et al., 2011; Wilfart et al., 2007). Interaction with environmental microbiota during the retention period provides an opportunity for transient gut colonization (Robinson and Breed, 2025). This further explains why pigs and chickens have less environmental microbiota than cattle, sheep, and goats. Genera such as *Flavobacterium*, *Pseudomonas*, *Acinetobacter*, and *Chryseobacterium* could be considered as indicators of microbial environmental-animal transmission in this study.

The shared microbiota among chickens, goats, and pigs included members of the phyla Firmicutes, Actinobacteria, Bacteroidetes, and Proteobacteria, while the microbiota shared by cattle, goats, pigs, and sheep consisted of members from the phyla Firmicutes, Proteobacteria, Spirochaetes, Verrucomicrobia, and Euryarchaeota. All the animal hosts, including environmental samples, shared five genera: *Acinetobacter*, *Chryseobacterium*, *Flavobacterium*, *Pedobacter*, and *Pseudomonas*. Genera such as *Arthrobacter*, *Bacillus*, *Corynebacterium*, *Microbacterium*, *Nocardioides*, and *Rhodococcus* were shared between all the animal hosts and soil, while water samples shared *Comamonas* and *Sphingobacterium* with the animal hosts. Further comparison of the shared genera between soil and each animal host revealed 10 genera that were shared between soil and cattle (*Aquabacterium*, *Mycobacterium*, *Mycolicibacterium*, *Pseudoxanthomonas*), soil and pigs (*Brevilactibacter*, *Intrasporangium*, *Janibacter*, *Serinicoccus*), and soil and chicken (*Chryseolinea*, *Citricoccus*) samples. Moreover, the microbiota of cattle (*Dyadobacter*, *Janthinobacterium*, *Polaromonas*, *Rhodoferax*, *Sphingopyxis*, *Spirosoma*), pigs (*Polynucleobacter*), and chicken (*Shigella*) samples exclusively shared 6, 1, 1 genera with water, respectively. None of the identified genera were exceptionally shared between the goat and the environment or the sheep and the environmental samples.

LEfSe analysis revealed host-adapted microbial genera that can be regarded as biomarkers in animal hosts and the environment. The biomarker genera in the water samples in this study were also found in high abundance in rivers near animal farms and in farm water in other studies (Abo-Ismail et al., 2024; Bai et al., 2019; Gu et al., 2022; Ke et al., 2023; Reza et al., 2018; Xu et al., 2024). Furthermore, the soil dwellers reported in this study were also found in high abundance in livestock farm soils by other researchers (Kilonzo-Nthenge et al., 2024; Męcik et al., 2023; Mhuireach et al., 2022; Yue et al., 2021; Zhang et al., 2025). The increased abundance of the reported environmental microbes, such as *Bradyrhizobium*, *Flavobacterium*, and *Streptomyces*, in soil and water was influenced by livestock farming (Męcik et al., 2023; Mhuireach et al., 2022; Xu et al., 2024). These environmentally adapted microbes are relevant to environmental health (Kharey et al., 2025; Mhuireach et al., 2022; Yue et al., 2021; Zhang et al., 2025).

The animal gut-adapted genera in sheep, pigs, chickens, goats, and cattle were also reported to be highly enriched in other studies, indicating their adaptation in the guts (Liu et al., 2021; Subrahmanyam et al., 2024; Wang et al., 2016, 2022, 2025; Zhang et al., 2022b). *Bacteroides*, *Treponema*, and *Prevotella*, which were highly enriched in sheep in this study, are implicated in the digestion of polysaccharides and oligosaccharides, lignocellulose, and hemicellulose, respectively (Cheng et al., 2022; Dao et al., 2021; Xie et al., 2018; Zafar and Saier Jr, 2021). *Acinetobacter*, which was highly abundant in sheep in this study, was shown to degrade a plant toxin via the steroid degradation pathway in a study by Li et al. (2022). *Rhodococcus*, highest in pigs, was found to be responsible for the degradation and utilization of plant polysaccharides (Yuan et al., 2020). Structural and intestinal barrier functions of *Clostridium* and *Solibacillus*, which were in high abundance in pigs of this study, were reported by other studies (Gao et al., 2025; Li et al., 2021c; Ostadmohammadi et al., 2022). Apart from opportunistic pathogens, some strains of *Enterococcus* and *Escherichia* have been shown to exhibit probiotic properties in livestock by modulating the gut environment to increase the abundance of beneficial bacteria such as *Lactobacillus*, thus improving intestinal health (Nakkarach et al., 2020; Shehata et al., 2020; Xie et al., 2018). *Ligilactobacillus animalis* has been reported to protect the intestinal epithelium, and *Ligilactobacillus agilis* has been shown to exhibit high antibacterial activity against pathogens, indicating the beneficial role of this genus in animal health (Boll et al., 2024; Yin et al., 2024). *Arthrobacter*, a highly enriched genus in cattle, was reported to be significantly enriched in the guts of yaks and a core genus in bar-headed geese, indicating its important role in gut microbial community structure (Wang et al., 2016, 2025). Johnson et al. (2006) reported xylose utilization and short-chain fatty acid production by *Arthrobacter* in the guts of Namaqua rock mice. *Brevundimonas* and *Comamonas* were part of the core gut microbiota of pathogen-resistant snails and silkworms raised under outdoor environmental conditions, highlighting the gut-adaptation and beneficial roles of these genera in the guts (Liu et al., 2025; Subrahmanyam et al., 2024). Furthermore, cellulolytic activity of *Brevundimonas vesicularis* was reported for the first time in a study by Hu et al. (2014), indicating the digestive role of this species in the gut. Although the reported genera were differentially abundant in each host, some similarities were observed at higher taxonomic ranks. Chickens and pigs in this study harbored higher levels of Firmicutes, bacteria that specialize in digesting starches and simple sugars, reflecting their diet, which often includes kitchen waste. These bacteria also support intestinal barrier function and gut health, highlighting how smallholder feeding practices foster beneficial microbial communities in monogastric hosts. In contrast, cattle, goats, and sheep, which graze on natural pastures, showed higher levels of Bacteroidetes, bacteria involved in breaking down complex plant polysaccharides and producing short-chain fatty acids for energy. The diversity and functions of their microbiomes are greatly influenced by environmental exposure, grazing management, and farm practices that determine access to soil, water, and vegetation. In support of this, genera of Alphaproteobacteria and Betaproteobacteria were differentially abundant in cattle and environmental samples, further underscoring how production systems and farm management shape gut microbial communities by controlling diet composition, environmental microbial exposure, and animal interactions, all of which together influence microbiome composition and functional potential. Overall, these results showed that the differentially abundant environmental and animal host-adapted microbial genera have commensal relationships with their hosts, which is crucial in the survival and resilience of these rural livestock and the ecosystem.

Pathogens were detected in lower abundance in these animals, but their presence remains a concern, as some are of clinical importance. The proximity of these animals to people of all ages poses potential health risks. Moreover, their unrestricted movement beyond household boundaries increases the likelihood of exposing more individuals to these pathogens. The low abundance of pathogens may suggest a balanced gut microbiome, where beneficial bacteria help suppress pathogens and maintain host health. The guts of free-range animals that survive in their natural environment have been reported to have low abundances of pathogens compared to animals that are kept in intensive systems (Chen et al., 2021a).

This study is the first to explore the gut microbiomes and the environmental microbiomes in smallholder mixed livestock farming systems in South Africa. These animals were all free-range, having an opportunity to explore various feed sources. From the findings, the microbiota is determined by the host, and the environment has some influence on microbial composition in livestock, especially ruminants. There is a notable interconnectedness within the ecosystem, as some environmental microbiota were found in the animal hosts. Additionally, the differences in microbiota among cattle, sheep, goats, pigs, and chickens appear to be influenced by their feeding behaviors, such as being ruminants or monogastric, as well as their feeding styles, which include grazing, browsing, and mixed feeding.

The application of metagenomics in this study facilitated comparative analyses of the gut microbiomes of rural livestock and their association with the environment, which is essential in the One Health framework. Shotgun metagenomic sequencing enables the assembly of metagenomes to reconstruct near-complete genomes of the organisms in the sample and predict their functional potential. Metagenomic sequencing of living animal or human gut microbiomes is facilitated by fecal sampling, which is non-invasive. Fecal sampling has been used as a proxy for gut microbiome analysis by several studies, as the microbiota in the feces represents the microbial communities in the gastrointestinal tract (Vogtmann et al., 2017). Also, fecal droppings are a primary route for gut microbial dissemination to the environment. As previously mentioned, the gut microbiomes of livestock from developing countries remain understudied, yet they also play a role in the overall health of these animals, which largely rely on natural vegetation for feed. Husbandry practices such as hygiene and biosecurity are important in protecting the health of animals; therefore, this calls for the need to educate farmers about the potential health risks that come with raising livestock in order to prevent the emergence of diseases from opportunistic pathogens.

## 5 Conclusion

Smallholder livestock farmers in rural systems often keep multiple types of animals in limited spaces, sharing grazing fields, water sources, and housing, typically under extensive production systems with varied farm management practices. Despite this, little is known about how livestock gut microbiomes interact with environmental microbial communities in these mixed-species systems. To address this knowledge gap, this study investigated the influence of mixed livestock farming systems on gut and environmental microbiomes in a smallholder rural context. Gut microbiomes were strongly influenced by host type and grazing behavior. Ruminants, particularly cattle, shared more microbial taxa with the environment compared to monogastrics. Significant differences were observed in both diversity and composition between soil and animal microbiomes, while water microbiomes were more similar to gut communities. Interestingly, while the microbiota found in soil and water samples differed from the microbiota present in the animals, there were shared microbial genera between these different environments. This sharing suggests potential transmission pathways for microbial communities, highlighting the importance of environmental interactions in shaping the microbiomes of livestock. Overall, these findings demonstrate that production systems, feeding behavior, and environmental exposure collectively shape livestock gut microbiomes in smallholder farming systems. It is noteworthy that the analysis was performed using sequencing reads before assembling them into contigs, thereby preserving the diversity that might be lost due to reads that cannot be assembled because of low or uneven sequencing depth. Therefore, future research should focus on the detailed characterization of these microbiomes to understand the functions of the microbial communities carried by these livestock and their potential roles in animal health. By identifying microbial signatures associated with resilience in livestock, researchers may uncover the specific adaptations that enable animals to thrive and effectively respond to various challenges faced in the smallholder livestock farming sector. This knowledge could ultimately inform better management practices and contribute to the sustainability of these farming systems.

## Data Availability

The data presented in the study are deposited in the NCBI Sequence Read Archive repository, accession number BioProject ID PRJNA1467375.
