# Supplementary material for: Metagenomic analysis of fecal and environmental microbiota in rural mixed livestock farming systems in South Africa
Source: Front Cell Infect Microbiol. 2026 Jul 9;16:1828785. doi: 10.3389/fcimb.2026.1828785 (PMC13391562; doi:10.3389/fcimb.2026.1828785)
Supplement: Supplementary file 1 [file Table1.docx]

Supplementary Material

# Supplementary Data

## Supplementary Figures

TM1


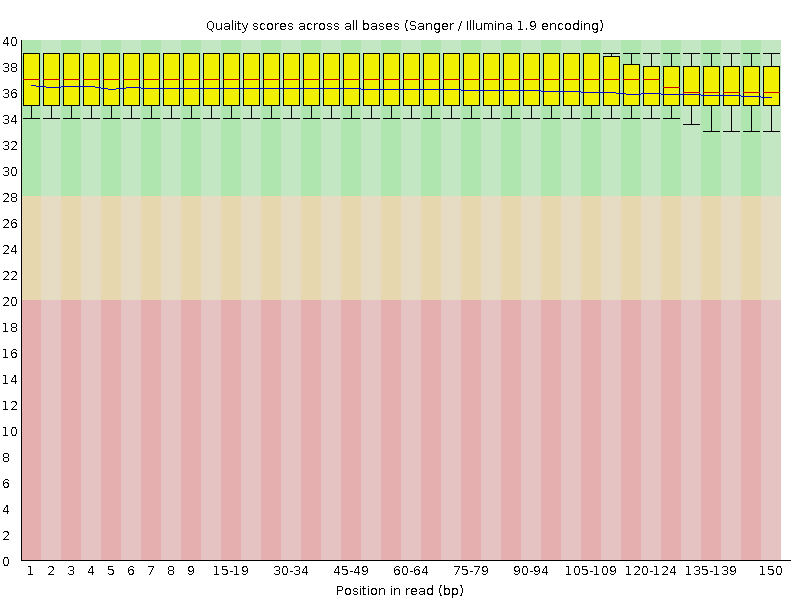

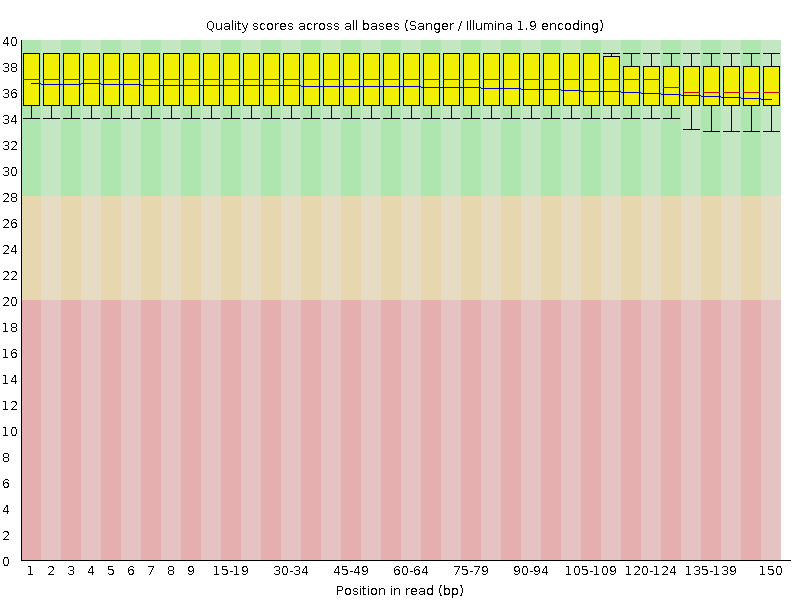


TM10


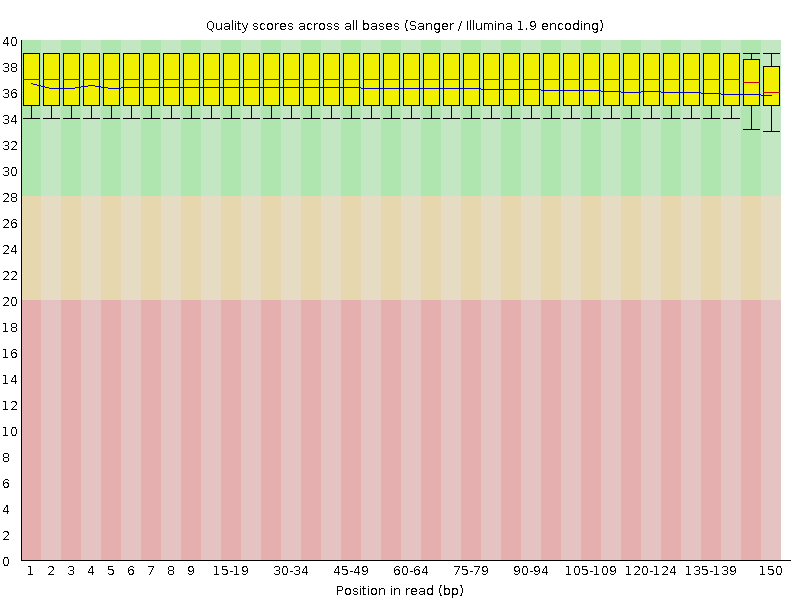

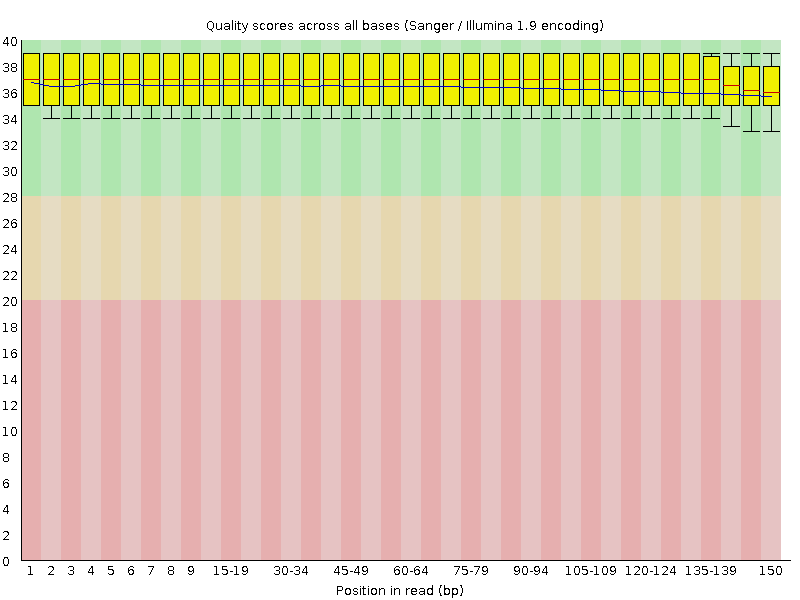


TM16


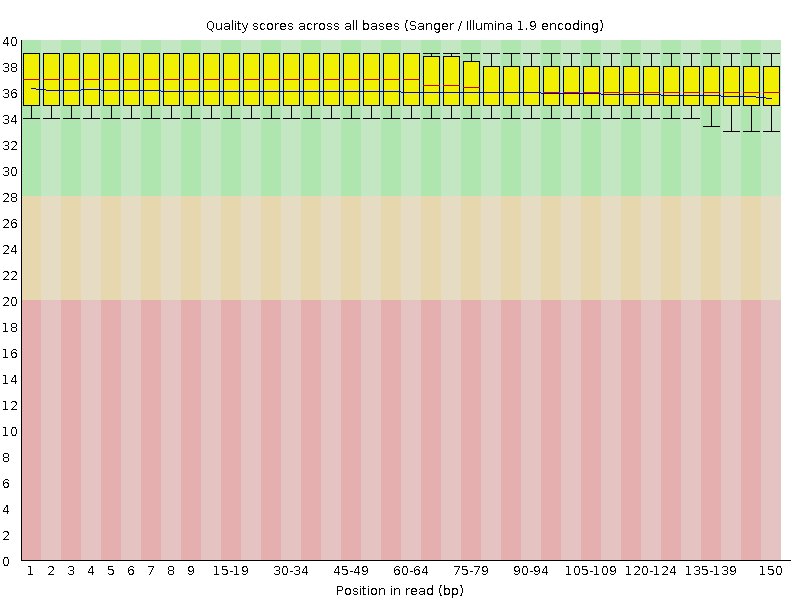

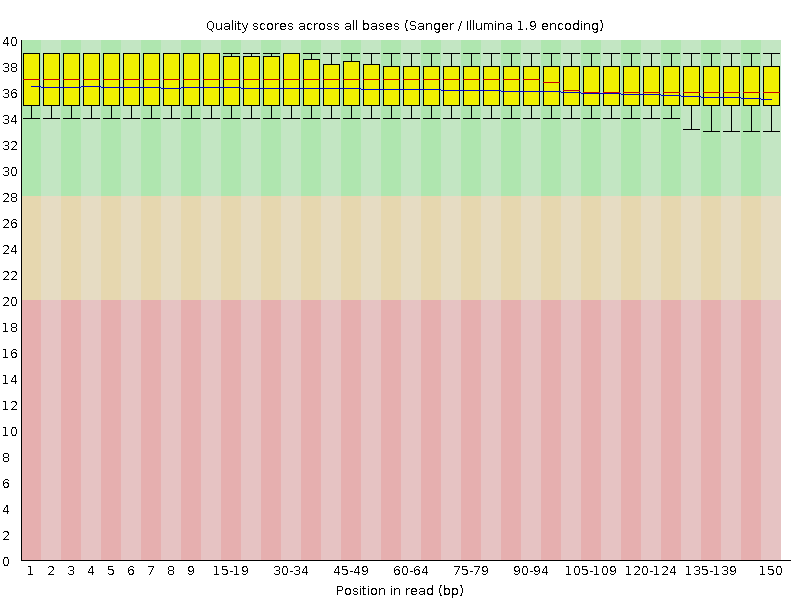


TM52


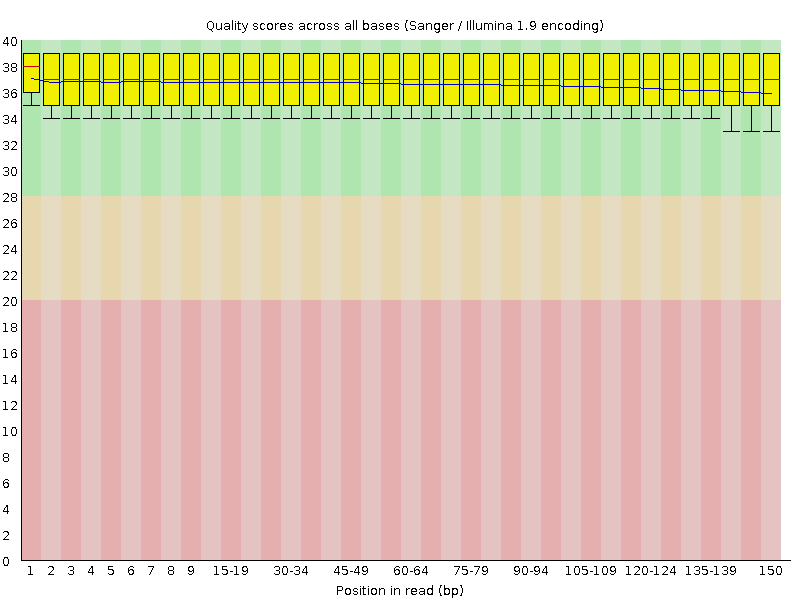

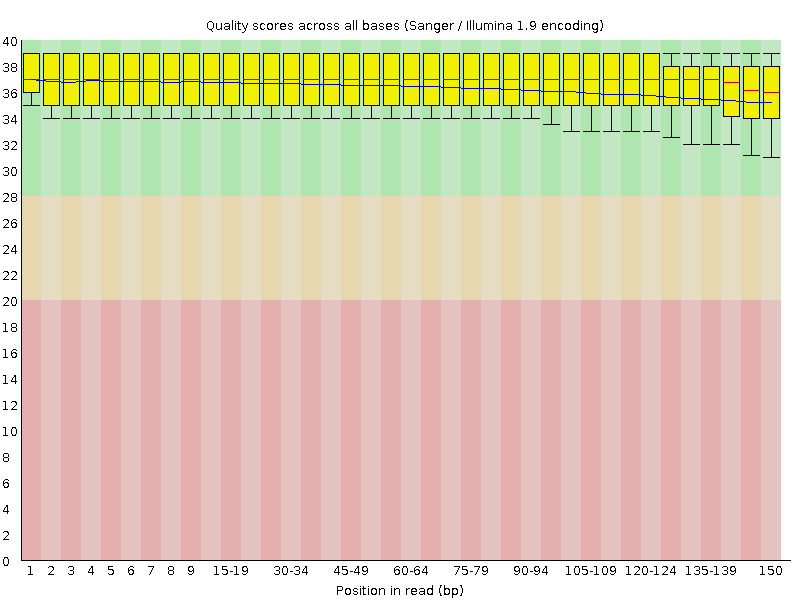


TM88


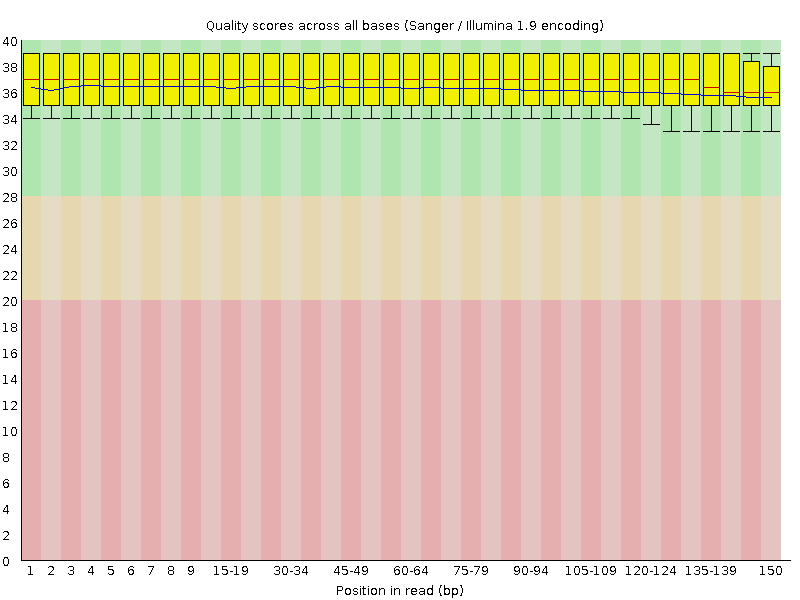

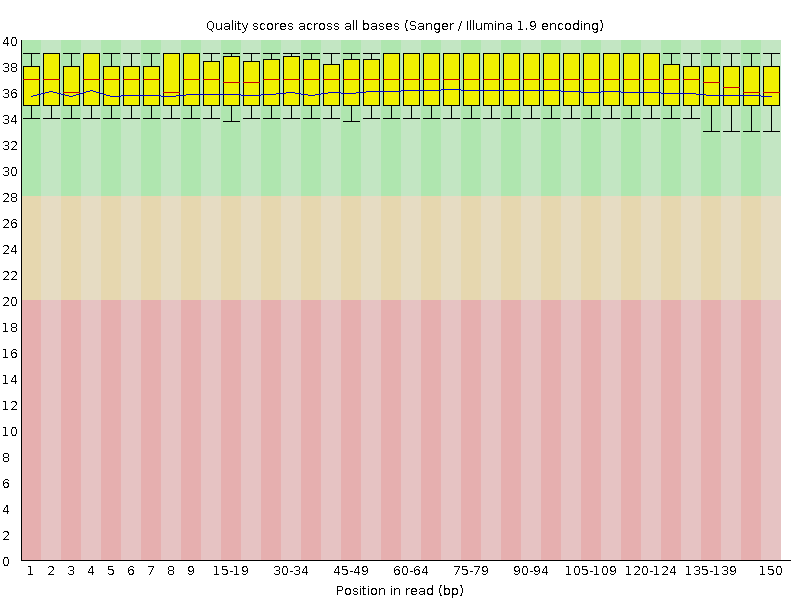


TM104


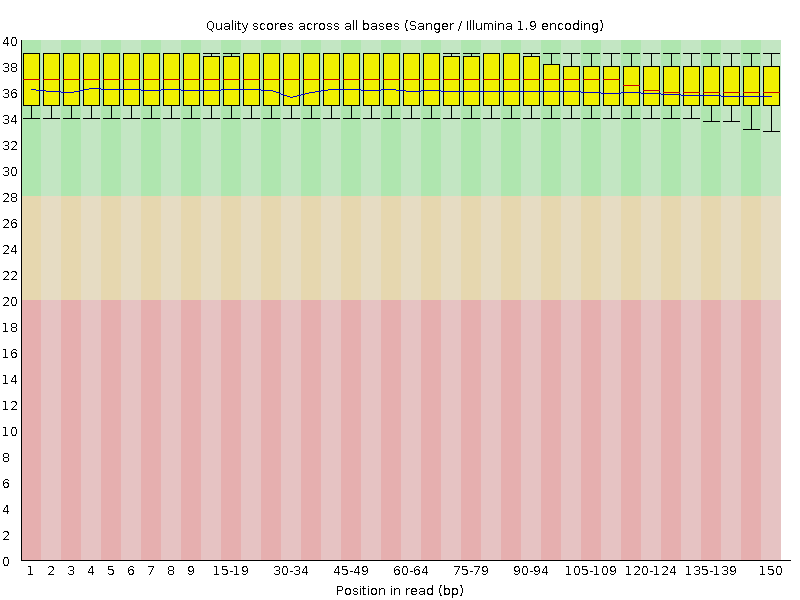

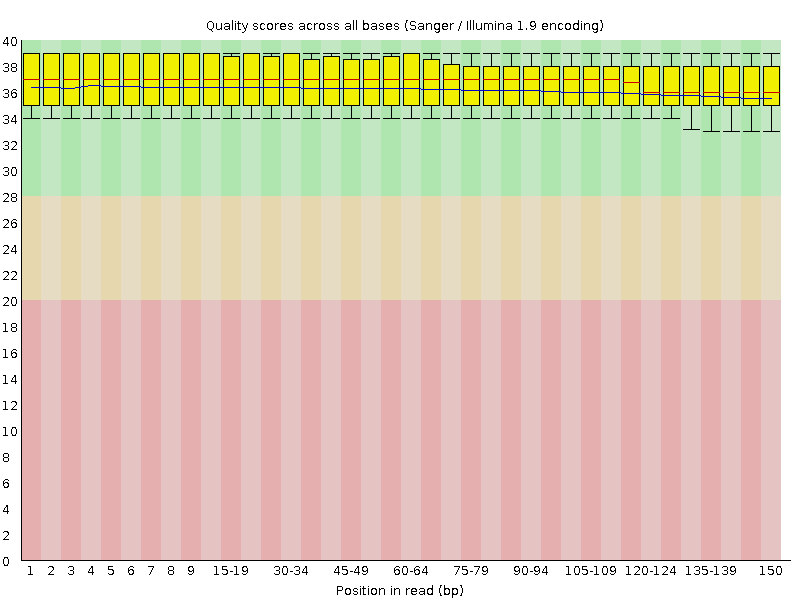


TM107


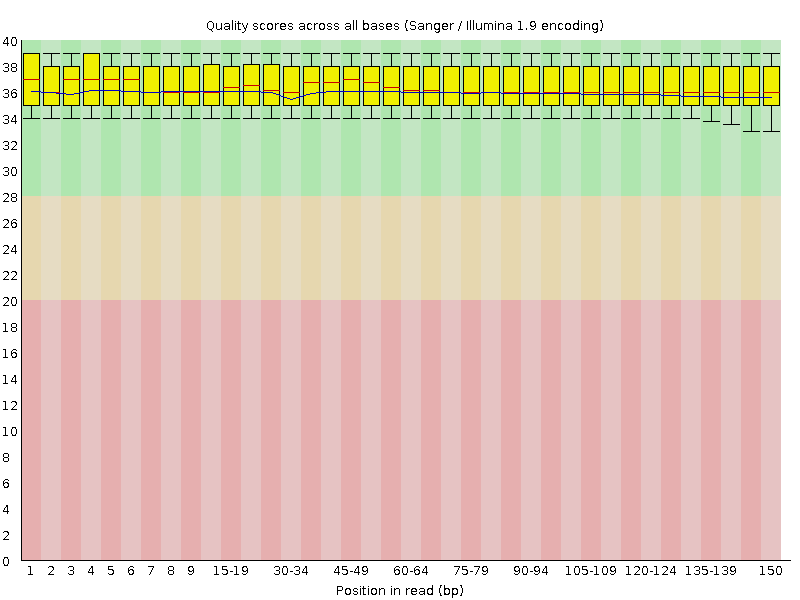

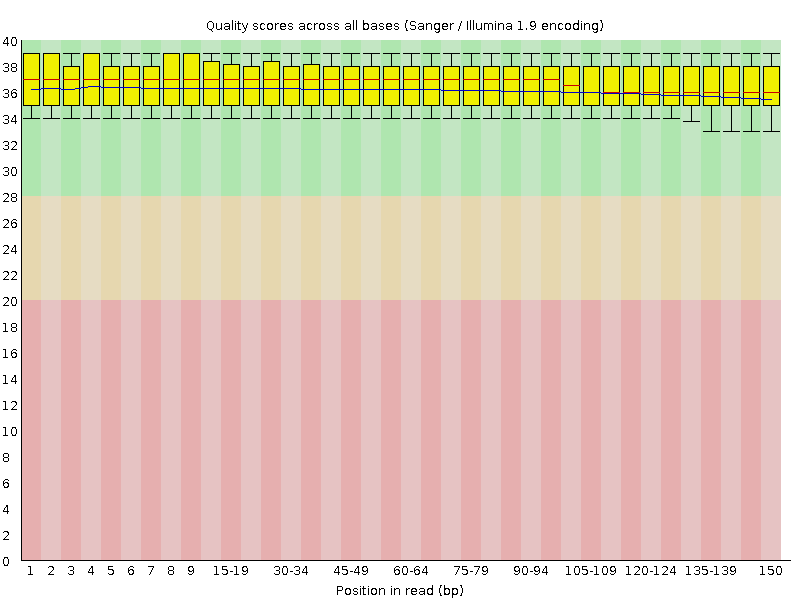


TM131


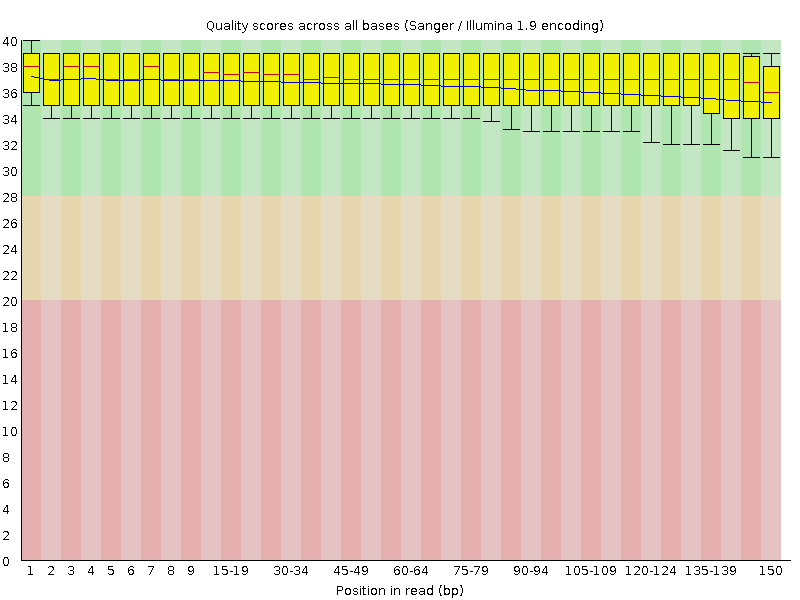

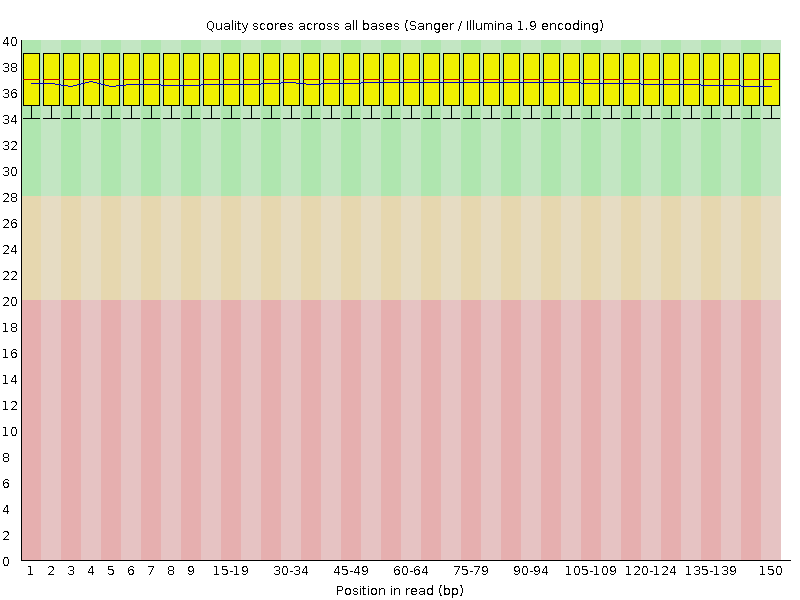


TM138


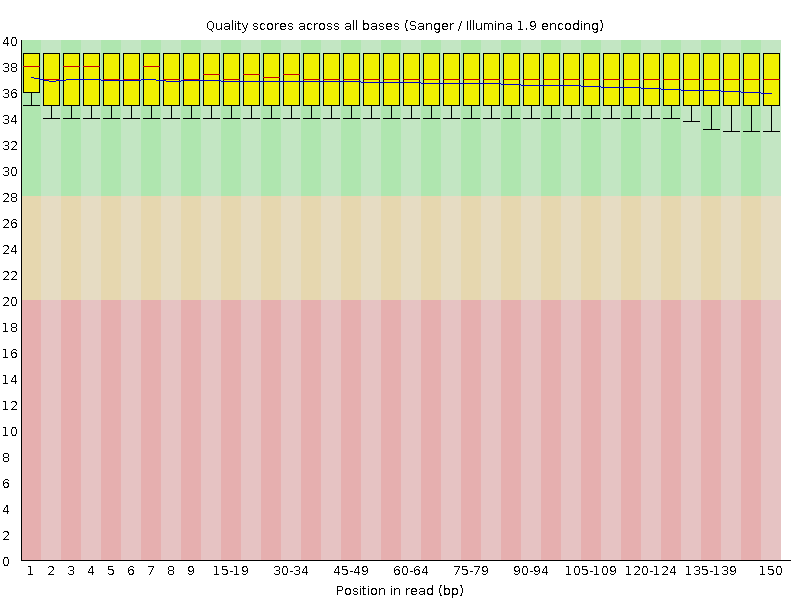

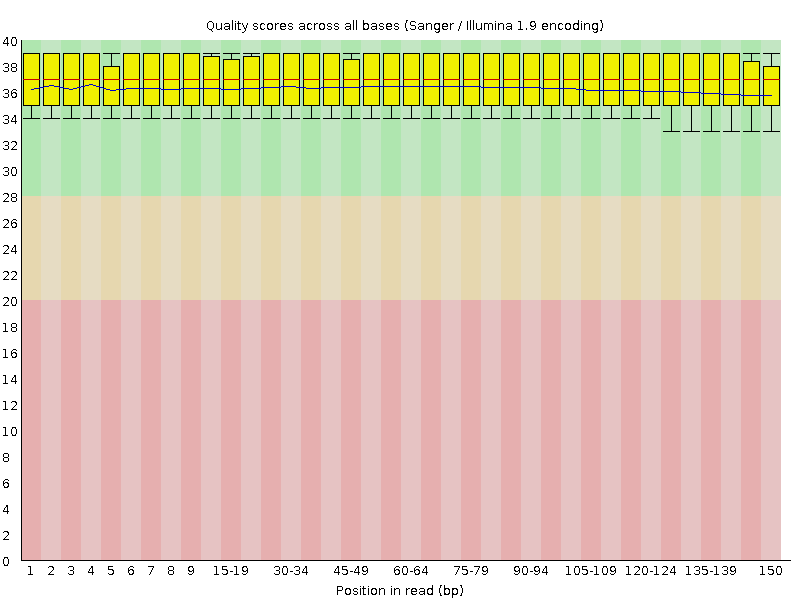


**Supplementary Figure 1**: FASTQC plots of representative samples showing the quality of the reads after sequencing (phred scores > 30). All the samples (111) had high quality reads therefore trimming was skipped.

TM1


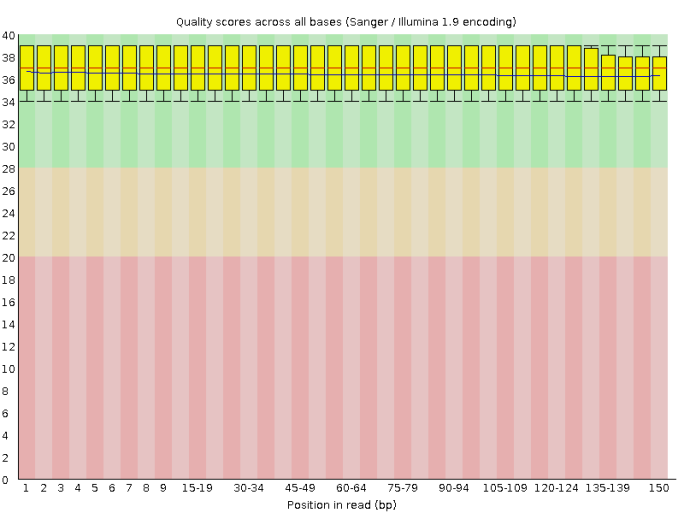

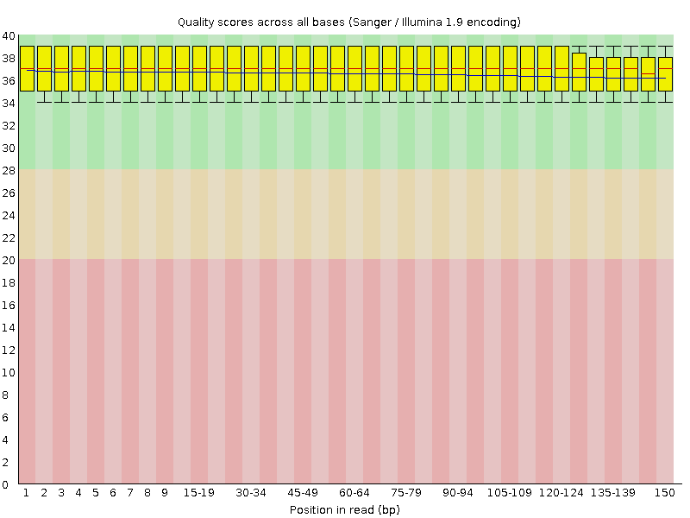


TM10


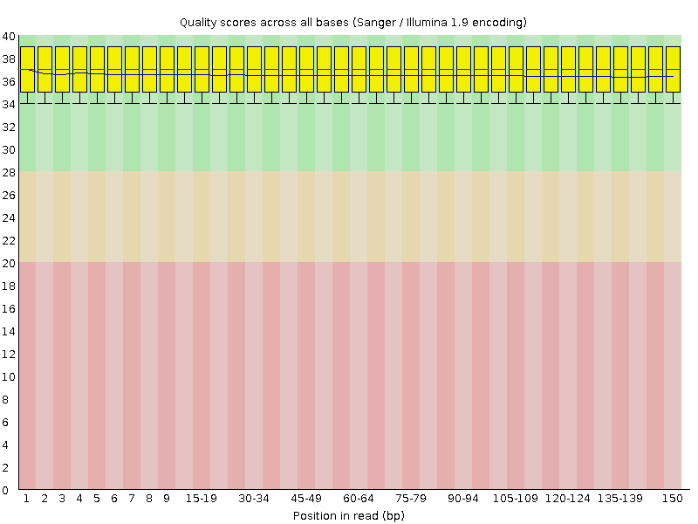

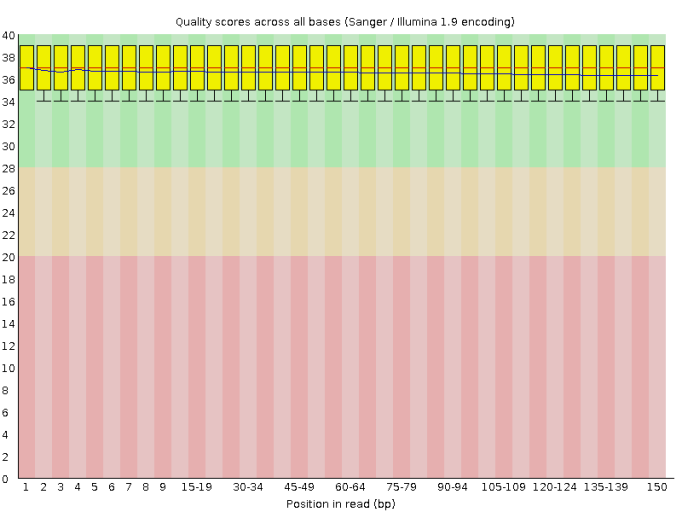


TM16


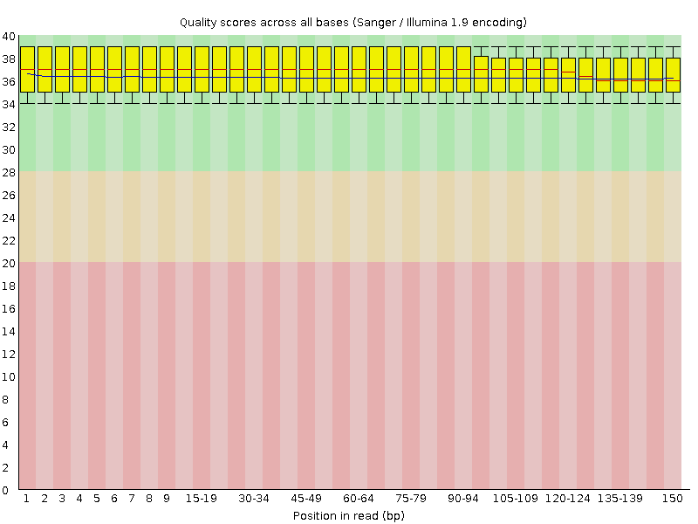

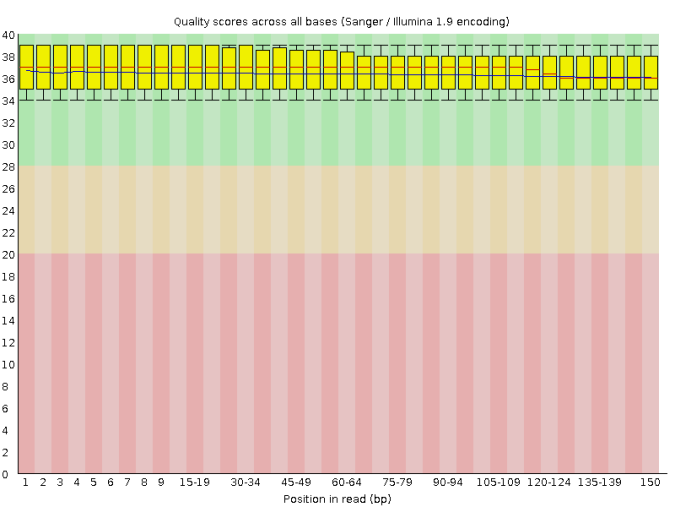


TM52


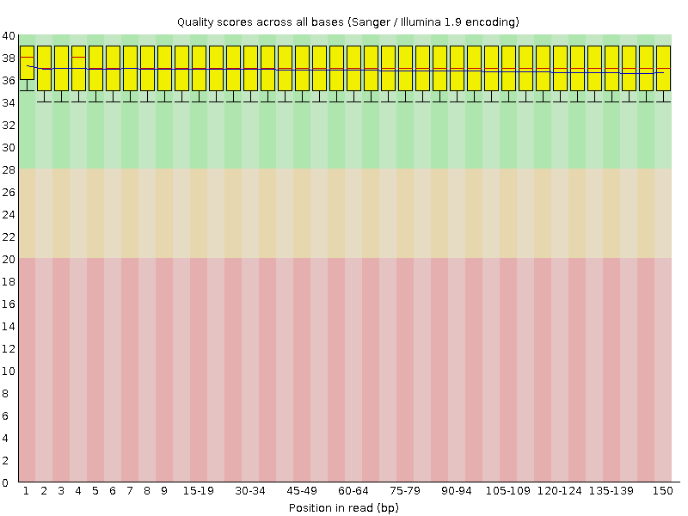

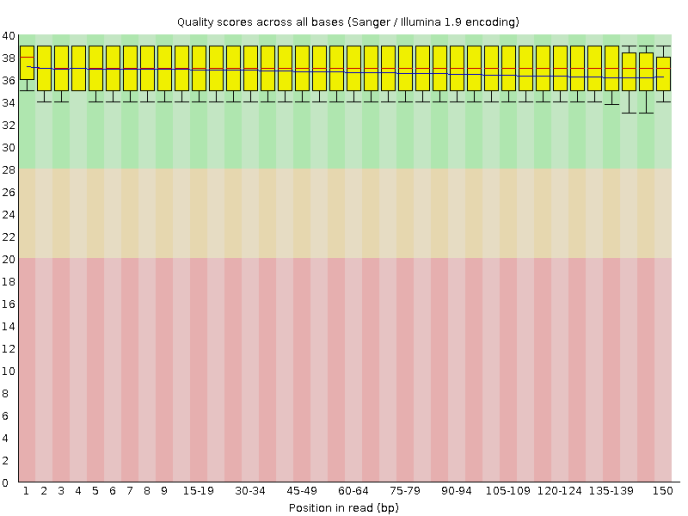


TM88


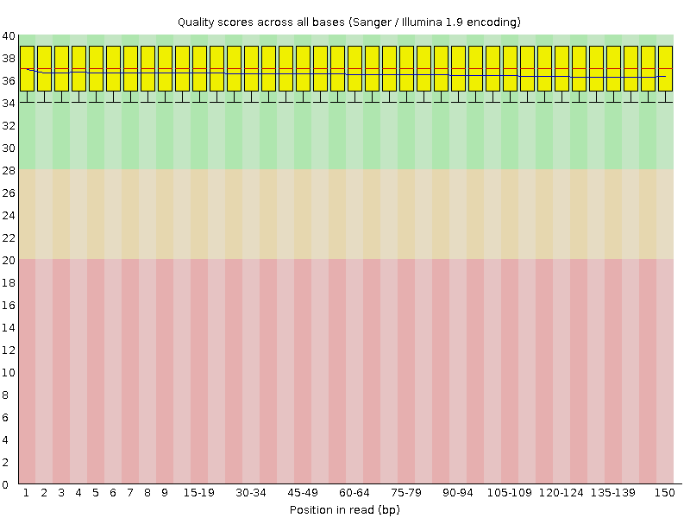

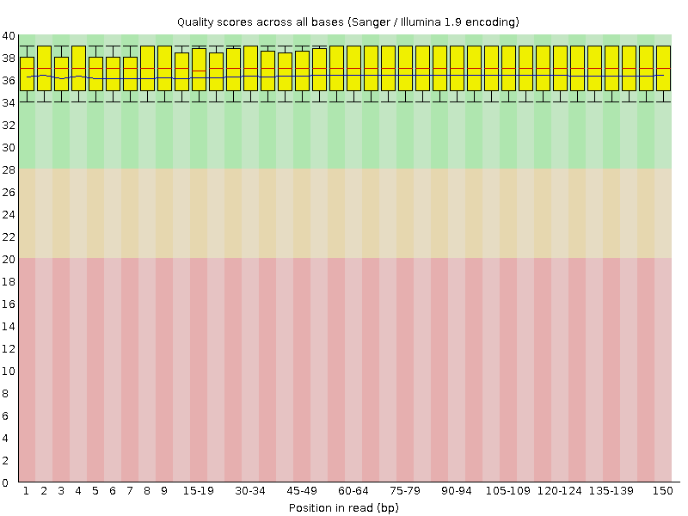


TM104


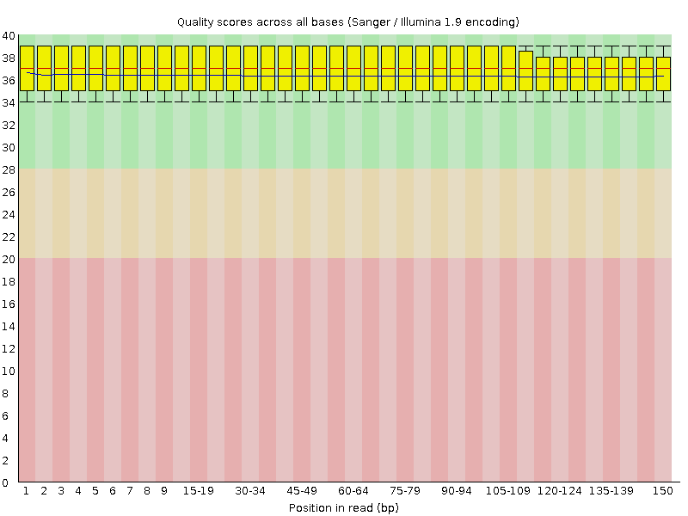

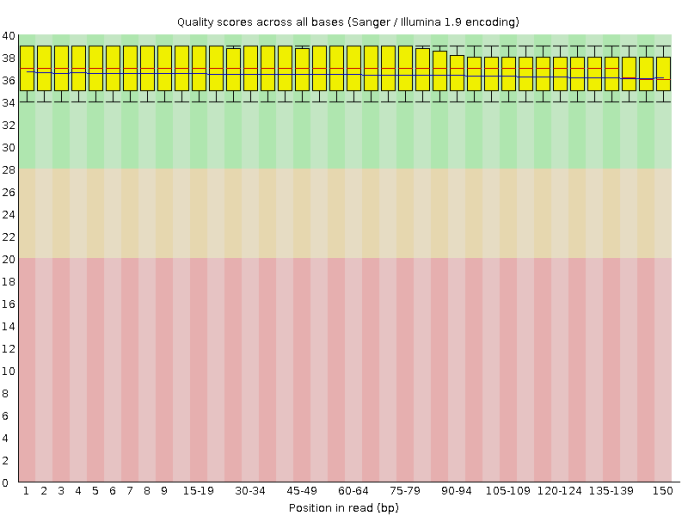


TM107


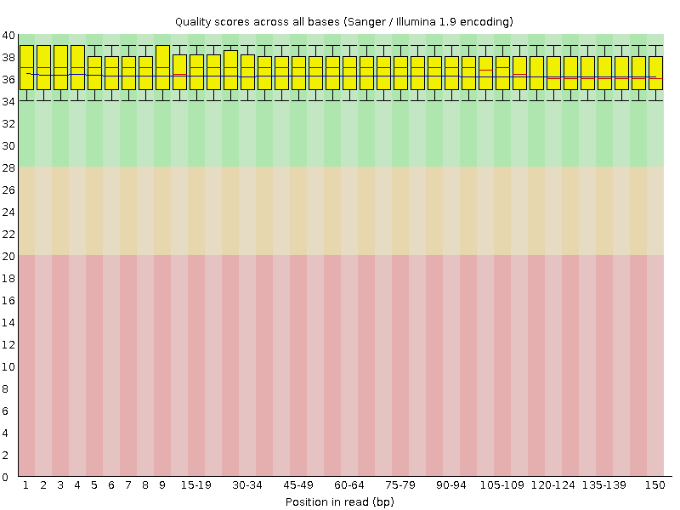

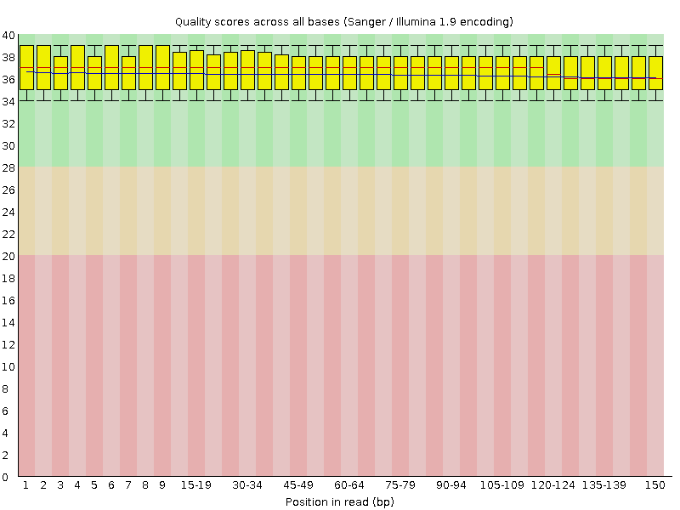


TM131


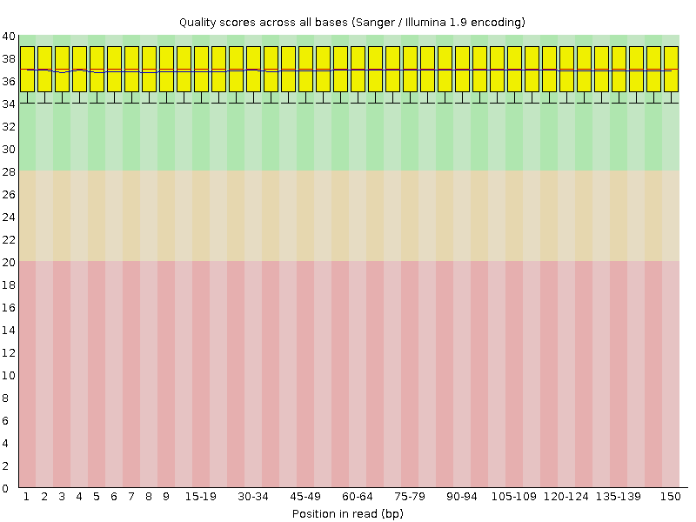

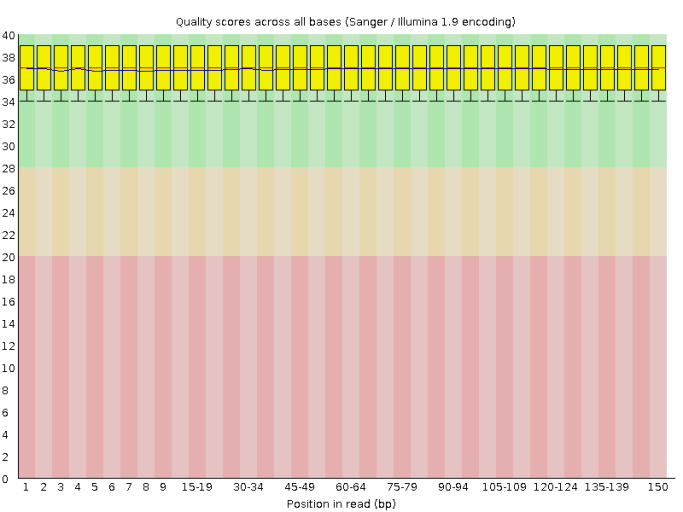


TM138


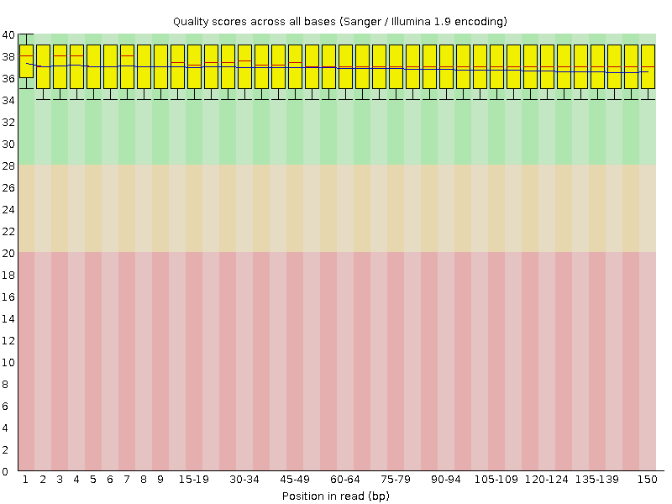

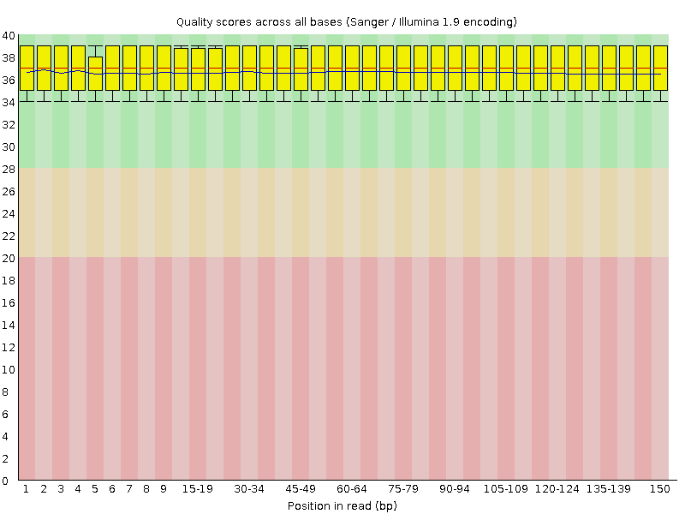


**Supplementary Figure 2**: FASTQC plots of representative samples showing the quality of the reads after adapter trimming, removal of low-quality reads, and read error correction using BBMap Version 38.95 (Bushnell, 2014).

## Supplementary Tables

Supplementary Table 1: Metadata of fecal, soil, and water samples collected from smallholder farming households in KwaZulu-Natal and Eastern Cape

| **Sample** | **Host** | **House** | **Province** | **Village** | **Feed** | **Water** |
| --- | --- | --- | --- | --- | --- | --- |
| TM1 | Cattle | H1 | Eastern Cape | Ncerha village 4 | Grazing | T |
| TM2 | Chicken | H1 | Eastern Cape | Ncerha village 4 | Grazing | T |
| TM3 | Goat | H1 | Eastern Cape | Ncerha village 4 | Grazing | T |
| TM4 | Pig | H1 | Eastern Cape | Ncerha village 4 | Grazing | T |
| TM5 | Cattle | H2 | Eastern Cape | Ncerha village 4 | Grazing, commercial | T, R |
| TM6 | Chicken | H2 | Eastern Cape | Ncerha village 4 | Grazing, commercial | T, R |
| TM7 | Goat | H2 | Eastern Cape | Ncerha village 4 | Grazing, commercial | T, R |
| TM8 | Pig | H2 | Eastern Cape | Ncerha village 4 | Grazing, commercial | T, R |
| TM9 | Cattle | H3 | Eastern Cape | Ncerha village 4 | Grazing, commercial | T |
| TM10 | Chicken | H3 | Eastern Cape | Ncerha village 4 | Grazing, commercial | T |
| TM11 | Goat | H3 | Eastern Cape | Ncerha village 4 | Grazing, commercial | T |
| TM12 | Pig | H3 | Eastern Cape | Ncerha village 4 | Grazing, commercial | T |
| TM13 | Cattle | H9 | Eastern Cape | Ncerha village 6 | Grazing, commercial | T |
| TM14 | Chicken | H9 | Eastern Cape | Ncerha village 6 | Grazing, commercial | T |
| TM15 | Goat | H9 | Eastern Cape | Ncerha village 6 | Grazing, commercial | T |
| TM16 | Pig | H9 | Eastern Cape | Ncerha village 6 | Grazing, commercial | T |
| TM17 | Cattle | H11 | Eastern Cape | Ncerha village 6 | Grazing, commercial | T |
| TM18 | Chicken | H11 | Eastern Cape | Ncerha village 6 | Grazing, commercial | T |
| TM19 | Goat | H11 | Eastern Cape | Ncerha village 6 | Grazing, commercial | T |
| TM20 | Pig | H11 | Eastern Cape | Ncerha village 6 | Grazing, commercial | T |
| TM21 | Cattle | H13 | Eastern Cape | Ncerha village 6 | Grazing | T |
| TM22 | Chicken | H13 | Eastern Cape | Ncerha village 6 | Grazing | T |
| TM23 | Goat | H13 | Eastern Cape | Ncerha village 6 | Grazing | T |
| TM24 | Pig | H13 | Eastern Cape | Ncerha village 6 | Grazing | T |
| TM25 | Cattle | H19 | Eastern Cape | Ncerha village 5 | Grazing | T |
| TM26 | Chicken | H19 | Eastern Cape | Ncerha village 5 | Grazing | T |
| TM27 | Goat | H19 | Eastern Cape | Ncerha village 5 | Grazing | T |
| TM28 | Pig | H19 | Eastern Cape | Ncerha village 5 | Grazing | T |
| TM29 | Cattle | H20 | Eastern Cape | Ncerha village 5 | Grazing | T |
| TM30 | Chicken | H20 | Eastern Cape | Ncerha village 5 | Grazing | T |
| TM31 | Goat | H20 | Eastern Cape | Ncerha village 5 | Grazing | T |
| TM32 | Pig | H20 | Eastern Cape | Ncerha village 5 | Grazing | T |
| TM33 | Cattle | H22 | Eastern Cape | Ncerha village 5 | Grazing | T |
| TM34 | Chicken | H22 | Eastern Cape | Ncerha village 5 | Grazing | T |
| TM35 | Goat | H22 | Eastern Cape | Ncerha village 5 | Grazing | T |
| TM36 | Pig | H22 | Eastern Cape | Ncerha village 5 | Grazing | T |
| TM50 | Soil | H1 | Eastern Cape | Ncerha village 4 | Soil | Soil |
| TM51 | Soil | H2 | Eastern Cape | Ncerha village 4 | Soil | Soil |
| TM52 | Soil | H3 | Eastern Cape | Ncerha village 4 | Soil | Soil |
| TM53 | Soil | H9 | Eastern Cape | Ncerha village 6 | Soil | Soil |
| TM54 | Soil | H11 | Eastern Cape | Ncerha village 6 | Soil | Soil |
| TM55 | Soil | H13 | Eastern Cape | Ncerha village 6 | Soil | Soil |
| TM56 | Soil | H19 | Eastern Cape | Ncerha village 5 | Soil | Soil |
| TM57 | Soil | H20 | Eastern Cape | Ncerha village 5 | Soil | Soil |
| TM58 | Soil | H22 | Eastern Cape | Ncerha village 5 | Soil | Soil |
| TM59 | Water | H1 | Eastern Cape | Ncerha village 4 | Water | Water |
| TM60 | Water | H2 | Eastern Cape | Ncerha village 4 | Water | Water |
| TM61 | Water | H3 | Eastern Cape | Ncerha village 4 | Water | Water |
| TM62 | Water | H9 | Eastern Cape | Ncerha village 6 | Water | Water |
| TM63 | Water | H11 | Eastern Cape | Ncerha village 6 | Water | Water |
| TM64 | Water | H13 | Eastern Cape | Ncerha village 6 | Water | Water |
| TM65 | Water | H19 | Eastern Cape | Ncerha village 5 | Water | Water |
| TM66 | Water | H20 | Eastern Cape | Ncerha village 5 | Water | Water |
| TM67 | Water | H22 | Eastern Cape | Ncerha village 5 | Water | Water |
| TM68 | Cattle | H35 | KwaZulu-Natal | Ndwebu | Grazing, commercial | T, R |
| TM69 | Goat | H35 | KwaZulu-Natal | Ndwebu | Grazing, commercial | T, R |
| TM70 | Chicken | H35 | KwaZulu-Natal | Ndwebu | Grazing, commercial | T, R |
| TM71 | Goat | H36 | KwaZulu-Natal | Ndwebu | Grazing | T, R |
| TM72 | Chicken | H36 | KwaZulu-Natal | Ndwebu | Grazing | T, R |
| TM73 | Pig | H36 | KwaZulu-Natal | Ndwebu | Grazing | T, R |
| TM74 | Goat | H37 | KwaZulu-Natal | Ndwebu | Grazing, commercial | T |
| TM75 | Chicken | H37 | KwaZulu-Natal | Ndwebu | Grazing, commercial | T |
| TM76 | Pig | H37 | KwaZulu-Natal | Ndwebu | Grazing, commercial | T |
| TM77 | Cattle | H38 | KwaZulu-Natal | Ndwebu | Grazing, commercial | T, R |
| TM78 | Goat | H38 | KwaZulu-Natal | Ndwebu | Grazing, commercial | T, R |
| TM79 | Chicken | H38 | KwaZulu-Natal | Ndwebu | Grazing, commercial | T, R |
| TM84 | Cattle | H40 | KwaZulu-Natal | KwaNokweja | Grazing | T, R |
| TM85 | Sheep | H40 | KwaZulu-Natal | KwaNokweja | Grazing | T, R |
| TM86 | Chicken | H40 | KwaZulu-Natal | KwaNokweja | Grazing | T, R |
| TM87 | Goat | H40 | KwaZulu-Natal | KwaNokweja | Grazing | T, R |
| TM88 | Pig | H40 | KwaZulu-Natal | KwaNokweja | Grazing | T, R |
| TM89 | Sheep | H41 | KwaZulu-Natal | KwaNokweja | Grazing, crop residue | T, R |
| TM90 | Goat | H41 | KwaZulu-Natal | KwaNokweja | Grazing, crop residue | T, R |
| TM92 | Pig | H41 | KwaZulu-Natal | KwaNokweja | Grazing, crop residue | T, R |
| TM96 | Chicken | H42 | KwaZulu-Natal | KwaNokweja | Grazing | T |
| TM97 | Pig | H42 | KwaZulu-Natal | KwaNokweja | Grazing | T |
| TM98 | Sheep | H43 | KwaZulu-Natal | Emazabekweni | Grazing | T, R |
| TM99 | Goat | H43 | KwaZulu-Natal | Emazabekweni | Grazing | T, R |
| TM100 | Chicken | H43 | KwaZulu-Natal | Emazabekweni | Grazing | T, R |
| TM102 | Cattle | H44 | KwaZulu-Natal | Emazabekweni | Grazing | T, R |
| TM103 | Sheep | H44 | KwaZulu-Natal | Emazabekweni | Grazing | T, R |
| TM104 | Goat | H44 | KwaZulu-Natal | Emazabekweni | Grazing | T, R |
| TM105 | Chicken | H44 | KwaZulu-Natal | Emazabekweni | Grazing | T, R |
| TM106 | Cattle | H45 | KwaZulu-Natal | Emazabekweni | Grazing | T, R |
| TM107 | Sheep | H45 | KwaZulu-Natal | Emazabekweni | Grazing | T, R |
| TM108 | Goat | H45 | KwaZulu-Natal | Emazabekweni | Grazing | T, R |
| TM109 | Chicken | H45 | KwaZulu-Natal | Emazabekweni | Grazing | T, R |
| TM110 | Cattle | H46 | KwaZulu-Natal | Emazabekweni | Grazing | T |
| TM111 | Chicken | H46 | KwaZulu-Natal | Emazabekweni | Grazing | T |
| TM112 | Pig | H46 | KwaZulu-Natal | Emazabekweni | Grazing | T |
| TM113 | Sheep | H1 | Eastern Cape | Ncerha village 4 | Grazing | T |
| TM114 | Sheep | H2 | Eastern Cape | Ncerha village 4 | Grazing, commercial | T, R |
| TM115 | Sheep | H3 | Eastern Cape | Ncerha village 4 | Grazing, commercial | T |
| TM116 | Pig | H4 | Eastern Cape | Ncerha village 4 | Grazing | T |
| TM117 | Soil | H35 | KwaZulu-Natal | Ndwebu | Soil | Soil |
| TM118 | Soil | H36 | KwaZulu-Natal | Ndwebu | Soil | Soil |
| TM119 | Soil | H37 | KwaZulu-Natal | Ndwebu | Soil | Soil |
| TM120 | Soil | H38 | KwaZulu-Natal | Ndwebu | Soil | Soil |
| TM121 | Soil | H39 | KwaZulu-Natal | KwaNokweja | Soil | Soil |
| TM123 | Soil | H41 | KwaZulu-Natal | KwaNokweja | Soil | Soil |
| TM124 | Soil | H42 | KwaZulu-Natal | KwaNokweja | Soil | Soil |
| TM127 | Soil | H45 | KwaZulu-Natal | Emazabekweni | Soil | Soil |
| TM128 | Soil | H46 | KwaZulu-Natal | Emazabekweni | Soil | Soil |
| TM130 | Water | H36 | KwaZulu-Natal | Ndwebu | Water | Water |
| TM131 | Water | H37 | KwaZulu-Natal | Ndwebu | Water | Water |
| TM133 | Water | H39 | KwaZulu-Natal | KwaNokweja | Water | Water |
| TM134 | Water | H40 | KwaZulu-Natal | KwaNokweja | Water | Water |
| TM135 | Water | H41 | KwaZulu-Natal | KwaNokweja | Water | Water |
| TM137 | Water | H43 | KwaZulu-Natal | Emazabekweni | Water | Water |
| TM138 | Water | H44 | KwaZulu-Natal | Emazabekweni | Water | Water |
| TM139 | Water | H45 | KwaZulu-Natal | Emazabekweni | Water | Water |

T: Tap water

R: River

Supplementary Table 5: Kruskal-Wallis multiple comparison p-values adjusted with the Benjamini-Hochberg method extracted from R to compare the microbial diversity differences between hosts.

**FISHER**

| **Comparison Z P.unadj P.adj** |
| --- |
| 1 Cattle - Chicken 2.56782795 1.023379e-02 3.070138e-02* |
| 2 Cattle - Goat -1.33223032 1.827845e-01 2.558983e-01 |
| 3 Chicken - Goat -4.11247808 3.914346e-05 4.110064e-04* |
| 4 Cattle - Pig 0.36677064 7.137901e-01 7.889259e-01 |
| 5 Chicken - Pig -2.22539697 2.605460e-02 6.079407e-02 |
| 6 Goat - Pig 1.73917878 8.200332e-02 1.565518e-01 |
| 7 Cattle - Sheep 0.34625363 7.291521e-01 7.656097e-01 |
| 8 Chicken - Sheep -1.74470093 8.103694e-02 1.701776e-01 |
| 9 Goat - Sheep 1.45284550 1.462667e-01 2.194000e-01 |
| 10 Pig - Sheep 0.04566258 9.635792e-01 9.635792e-01 |
| 11 Cattle - Soil -2.76911834 5.620822e-03 2.360745e-02* |
| 12 Chicken - Soil -5.54693075 2.907278e-08 6.105284e-07* |
| 13 Goat - Soil -1.54096355 1.233257e-01 2.158199e-01 |
| 14 Pig - Soil -3.18772536 1.433967e-03 7.528325e-03* |
| 15 Sheep - Soil -2.64842944 8.086673e-03 2.830336e-02* |
| 16 Cattle - Water 1.06815872 2.854489e-01 3.746517e-01 |
| 17 Chicken - Water -1.52322200 1.277032e-01 2.062897e-01 |
| 18 Goat - Water 2.49598795 1.256069e-02 3.297180e-02* |
| 19 Pig - Water 0.70790580 4.790038e-01 5.917105e-01 |
| `  20 Sheep - Water 0.52898845 5.968135e-01 6.962824e-01 |
| 21 Soil - Water 3.94356957 8.027769e-05 5.619438e-04* |

*Significant (P < 0.05)

**OBSERVED**

| **Comparison Z P.unadj P.adj** |
| --- |
| 1 Cattle - Chicken 2.725691790 6.416689e-03 2.695009e-02* |
| 2 Cattle - Goat -1.362123431 1.731589e-01 2.597384e-01 |
| 3 Chicken - Goat -4.310023364 1.632373e-05 1.713992e-04* |
| 4 Cattle - Pig 0.369490360 7.117623e-01 7.866846e-01 |
| 5 Chicken - Pig -2.383211271 1.716234e-02 5.148702e-02 |
| 6 Goat - Pig 1.772439678 7.632160e-02 1.457049e-01 |
| 7 Cattle - Sheep 0.305583020 7.599222e-01 7.979183e-01 |
| 8 Chicken - Sheep -1.916319530 5.532444e-02 1.161813e-01 |
| 9 Goat - Sheep 1.435536824 1.511342e-01 2.441398e-01 |
| 10 Pig - Sheep 0.002285205 9.981767e-01 9.981767e-01 |
| 11 Cattle - Soil -2.312205696 2.076635e-02 5.451167e-02 |
| 12 Chicken - Soil -5.223663888 1.754171e-07 3.683758e-06* |
| 13 Goat - Soil -1.032616508 3.017834e-01 3.960907e-01 |
| 14 Pig - Soil -2.726024614 6.410222e-03 3.365366e-02* |
| 15 Sheep - Soil -2.228074982 2.587552e-02 6.037620e-02 |
| 16 Cattle - Water 1.047396973 2.949165e-01 4.128831e-01 |
| 17 Chicken - Water -1.708576103 8.752950e-02 1.531766e-01 |
| 18 Goat - Water 2.505142174 1.224022e-02 4.284076e-02* |
| 19 Pig - Water 0.683984292 4.939851e-01 6.102169e-01 |
| 20 Sheep - Water 0.553363521 5.800145e-01 6.766836e-01 |
| 21 Soil - Water 3.451004057 5.585052e-04 3.909536e-03* |

*Significant (P < 0.05)

**SIMPSON**

| **Comparison Z P.unadj P.adj** |
| --- |
| 1 Cattle - Chicken 2.5710418 1.013931e-02 3.041793e-02* |
| 2 Cattle - Goat -0.3720190 7.098787e-01 7.846028e-01 |
| 3 Chicken - Goat -3.0952564 1.966429e-03 1.032375e-02* |
| 4 Cattle - Pig 1.0306255 3.027165e-01 4.238031e-01 |
| 5 Chicken - Pig -1.5255133 1.271311e-01 2.669753e-01 |
| 6 Goat - Pig 1.4565622 1.452373e-01 2.772711e-01 |
| 7 Cattle - Sheep 0.6292783 5.291669e-01 6.536767e-01 |
| 8 Chicken - Sheep -1.4533413 1.461290e-01 2.557258e-01 |
| 9 Goat - Sheep 0.9544316 3.398652e-01 4.460731e-01 |
| 10 Pig - Sheep -0.2191804 8.265095e-01 8.265095e-01 |
| 11 Cattle - Soil -2.7874569 5.312353e-03 1.859323e-02* |
| 12 Chicken - Soil -5.5696264 2.552862e-08 5.361010e-07* |
| 13 Goat - Soil -2.5371549 1.117575e-02 2.933634e-02* |
| 14 Pig - Soil -3.8811954 1.039443e-04 7.276098e-04* |
| 15 Sheep - Soil -2.9498024 3.179772e-03 1.335504e-02* |
| 16 Cattle - Water 1.3798509 1.676325e-01 2.707910e-01 |
| 17 Chicken - Water -1.1958114 2.317702e-01 3.476552e-01 |
| 18 Goat - Water 1.8298817 6.726763e-02 1.569578e-01 |
| 19 Pig - Water 0.3399284 7.339105e-01 7.706060e-01 |
| 20 Sheep - Water 0.4975204 6.188221e-01 7.219591e-01 |
| 21 Soil - Water 4.2794909 1.873213e-05 1.966874e-04* |

*Significant (P < 0.05)

**SHANNON**

| **Comparison Z P.unadj P.adj** |
| --- |
| 1 Cattle - Chicken 2.6359606 8.389948e-03 2.516984e-02* |
| 2 Cattle - Goat -0.7460490 4.556378e-01 5.628467e-01 |
| 3 Chicken - Goat -3.5609791 3.694745e-04 1.939741e-03* |
| 4 Cattle - Pig 0.8703467 3.841109e-01 5.041456e-01 |
| 5 Chicken - Pig -1.7613637 7.817687e-02 1.641714e-01 |
| 6 Goat - Pig 1.6694838 9.502155e-02 1.814048e-01 |
| 7 Cattle - Sheep 0.5654472 5.717696e-01 6.670645e-01 |
| 8 Chicken - Sheep -1.5728481 1.157540e-01 2.025695e-01 |
| 9 Goat - Sheep 1.1964002 2.315404e-01 3.241566e-01 |
| 10 Pig - Sheep -0.1506865 8.802230e-01 8.802230e-01 |
| 11 Cattle - Soil -2.9257294 3.436497e-03 1.202774e-02* |
| 12 Chicken - Soil -5.7821677 7.374413e-09 1.548627e-07* |
| 13 Goat - Soil -2.3012149 2.137949e-02 5.612116e-02 |
| 14 Pig - Soil -3.8588254 1.139333e-04 7.975329e-04* |
| 15 Sheep - Soil -3.0000313 2.699519e-03 1.133798e-02* |
| 16 Cattle - Water 1.3731968 1.696912e-01 2.741165e-01 |
| 17 Chicken - Water -1.2700362 2.040717e-01 3.061076e-01 |
| 18 Goat - Water 2.2095531 2.713619e-02 6.331779e-02 |
| 19 Pig - Water 0.4985393 6.181040e-01 6.490092e-01 |
| 20 Sheep - Water 0.5572012 5.773900e-01 6.381679e-01 |
| 21 Soil - Water 4.4153955 1.008255e-05 1.058668e-04* |

*Significant (P < 0.05)

**Reference**

Bushnell B. (2014). BBMap: a fast, accurate, splice-aware aligner.
